# A Baltic Sea microbiome-derived Type III polyketide synthase produces host-protective alkylresorcinols in *E. coli*

**DOI:** 10.1371/journal.pone.0361965

**Published:** 2026-11-03

**Authors:** Susana López-Gutiérrez, Laura A. Espinosa-Barrera, Arianna Soto-Hernández, Holber Zuleta-Prada, Celia Bustos-Brito, César S. Cardona-Félix, José Utrilla, Beatriz Velazquez-Cruz, Yair Cárdenas-Conejo, Jesus Alfredo Araujo-León, Victor Aguilar-Hernández, Juan A. Osuna-Castro, Baldomero Esquivel, Hugo Serrano-Posada, Sara Centeno-Leija

**Affiliations:** 1 Laboratorio de Biología Sintética, Estructural y Molecular, Facultad de Ingeniería Mecánica y Eléctrica, Universidad de Colima, Coquimatlán, Colima, México; 2 Unidad de Biología Integrativa, Centro de Investigación Científica de Yucatán, A. C. (CICY), Mérida, Yucatán, México; 3 Departamento de Microbiología Molecular, Instituto de Biotecnología, Universidad Nacional Autónoma de México, Cuernavaca, Morelos, México; 4 Laboratorio de Productos Naturales, Área de Química, Departamento de Preparatoria Agrícola, Universidad Autónoma Chapingo, Chapingo, México; 5 Instituto de Química, Universidad Nacional Autónoma de México, Ciudad Universitaria, Ciudad de México, México; 6 Secretaría de Ciencia, Humanidades, Tecnología e Innovación, Centro de Investigaciones Biológicas del Noroeste, La Paz, Baja California Sur, Mexico; 7 Centro de Ciencias Genómicas, Universidad Nacional Autónoma de México, Av. Universidad S/N Col. Chamilpa, Cuernavaca, Mor, México; 8 Facultad de Ciencias Químicas, Universidad de Colima, Coquimatlán, Colima, México; 9 Secretaría de Ciencia, Humanidades, Tecnología e Innovación, Laboratorio de Biología Sintética, Estructural y Molecular, Centro Universitario de Investigaciones Biomédicas (CUIB), Universidad de Colima, Colima, México; 10 Facultad de Ciencias Biológicas y Agropecuarias, Universidad de Colima, Tecomán, Colima, México; 11 Secretaría de Ciencia, Humanidades, Tecnología e Innovación, Laboratorio de Biología Sintética, Estructural y Molecular, Facultad de Ingeniería Mecánica y Eléctrica, Universidad de Colima, Coquimatlán, Colima, México; Yenepoya University, INDIA

## Abstract

Marine microorganisms are a prolific source of novel polyketide synthases (PKSs) that generate structurally diverse and functional polyketides. Here, we identify a novel type III PKS, ArsMB, from *Pontimonas* sp. via metagenomic mining of a Baltic Sea microbiome. Heterologous expression of *ars*MB synthetic gene in an *Escherichia coli* host that lacks a native type III PKS, combined with a two-step strategy to boost acyl-CoA precursor availability in a glucose-mineral medium, enabled the *in vivo* synthesis of two cell-associated alkylresorcinols, identified by GC-MS as 5-pentadecyl-1,3-benzenediol (ars1) and 5-tridecyl-1,3-benzenediol (ars2). The product profile is consistent with a tetraketide alkylresorcinol biosynthetic pathway utilizing C16 and C14 acyl starter units, drawn from the host acyl-CoA pool. In agreement, AlphaFold2-based structural modeling predicts a Cys139/His275/Asn308 catalytic triad and a long hydrophobic substrate tunnel compatible with long-chain acyl starter units. *E. coli* producing the amphipathic cell-associated alkylresorcinols ars1/ars2 exhibited increased resistance to muralytic digestion by endolysins and to hydrogen peroxide. These findings expand the known repertoire of marine-derived type III PKSs and highlight the biotechnological potential of ArsMB-derived alkylresorcinols.

## Introduction

Genome mining has emerged as a pivotal tool for uncovering novel secondary metabolites by targeting biosynthetic gene clusters (BGCs) within the “microbial dark matter” of uncharted marine ecosystems [1,2]. The Baltic Sea, a semi-enclosed brackish environment, offers a unique bioprospecting hotspot due to its distinct hydrography and vulnerability to anthropogenic stressors [3,4]. Microbial communities in this basin must adapt to the synergistic effects of physicochemical gradients, pollutant exposure, and intense bacteriophage predation [5,6], selective pressures that drive the evolution of sophisticated molecular defense systems and enzymatic versatility [7]. This unique ecological context serves as a reservoir of high-value biotechnological tools and bioactive compounds [8], evidenced by the isolation of the antiviral compound cyanopeptolin CP978 from *Nostoc edaphicum* [9], the development of whole-cell biosensors using *Vibrio harveyi* [10], as well as the discovery of functional traits related to bioplastic production [11], hydrocarbon remediation [12], and microbial population-controlling lytic viruses [13,14]. Among the bioactive metabolites derived from Baltic Sea microbiomes, polyketides constitute a significant class [15–17]. Notable examples include lindgomycin, an antibiotic polyketide isolated from a sponge-associated endophyte [15], as well as the macrolide balticolid and the isoindole conioimide, which display distinct antiviral and anti-inflammatory activities, respectively [16,17]. While structurally diverse, polyketides share a conserved biosynthetic mechanism catalyzed by polyketide synthases (PKSs) [18]. Unlike the multimeric type I and II systems, type III PKSs (PKSs III) are homodimeric enzymes (approximately 80–90 kDa) with a simpler architecture [19,20]. Typically, type III PKSs extend an initiating acyl-CoA substrate using malonyl-CoA, though many also accept acyl-acyl carrier protein (ACP) substrates from fatty acid biosynthesis [21]. Through iterative Claisen-type decarboxylative condensations, followed by intramolecular cyclization and aromatization, these enzymes generate diverse scaffolds, including chalcones, stilbenes, pyrones, and alkylresorcinols [22]. Alkylresorcinols (ARs) are bioactive phenolic lipids characterized by a 1, 3-dihydroxylated benzene ring (resorcinol core) with a hydrophobic aliphatic chain at the C5 position [23]. The amphiphilic nature of ARs allows them to integrate into lipid bilayers, modulating membrane architecture and physiology [23,24]. Prokaryotic type III PKSs dedicated to biosynthesis of amphipathic membrane-active ARs are often encoded in *srs*-like BGCs (*Streptomyces* alkylresorcinol synthases-like BGC) [25]. This cluster has been found in distinct phyla, including Actinomycetota [25,26], Cyanobacteriota [21], Planctomycetota [27], and Pseudomonadota [28]. During biosynthesis, ARs are incorporated into phospholipid bilayers, contributing to the lytic resistance of *Azotobacter vinelandii* and pathogenic species of *Mycobacterium* [29]. Furthermore, cytoplasmic membrane-associated ARs contribute to β-lactam antibiotic resistance in Actinobacteria [25]. Also, membrane-active ARs promote protective effects under stress conditions such as heat shock, oxidative stress, and radiation [30]. These protective effects are closely linked to their antioxidant activity, primarily attributed to the hydroxyl groups at positions C1-C3 on the benzene ring, which enable free radical scavenging through hydrogen donation [31,32]. In this sense, the high selection pressure exerted by stressor xenobiotics, intense ecological competition, and predation by membrane-lytic bacteriophages [5,33,34] makes the Baltic Sea microbiomes an ideal reservoir for bioprospecting novel alkylresorcinol-producing type III polyketide synthases.

In this study, we report the identification of a putative PKS III, designated ArsMB (NCBI ID: KRO38894.1), detected in the genome of uncultured *Pontimonas* sp via mining of Baltic Sea metagenomic data. Comparative genomic and phylogenetic analysis, along with 3D protein structure modeling, suggest that ArsMB is a novel PKS III involved in the synthesis of alkylresorcinols from long-chain acyl-CoA/ACP substrates. To validate this, we expressed a synthetic *ars*MB biological circuit in *E. coli*, a host that lacks a native type III PKS, combined with a two-step strategy to boost acyl-CoA precursor availability in a glucose-mineral medium. The isolation and characterization of the resulting amphipathic cell-associated alkylresorcinols, along with lytic-resistant tests using muralytic enzymes and lethal peroxide dosages, suggest potential biotechnological applications.

## Materials and methods

### Data mining

Metagenomes were analyzed from the Joint Genome Institute (JGI) IMG/M Database [35], which contains over 40,000 metagenomes from different environments (last search, December 2025). Putative bacterial type III PKSs were identified using a BLAST search in 390 publicly assembled metagenomes in the IMG/M platform with an E-value cutoff of 1.0e^-5^. The metagenomes were filtered to include those obtained from the Baltic Sea samples. The protein query sequence comprised the complete amino acid sequences of experimentally characterized PKSs III of crystal structures solved: the germicidin synthase (GCS) from *Streptomyces coelicolor* (NCBI ID: CAB94644.1, PDB ID: 3V7I) [36,37] and the alkylpyrone synthase (PKS11) from *Mycobacterium tuberculosis* (NCBI ID: P9WPF3.1, PDB ID: 4JAO) [38,39]. Putative type III PKSs sharing > 50% sequence identity with query sequences were excluded to increase novelty. The best hits were manually analyzed to discard truncated sequences. Functional domains of the selected hits were predicted using NCBI’s Batch Web CD-Search Tool against the Conserved Domain Database (CDD/SPARCLE) [40,41], Pfam [42], and UniProt (The Uniprot Consortium, 2023; https://www.uniprot.org/). The *ars*MB gene (JGI Gene ID Ga0633420_0034487_768_1892; NCBI Protein ID: KRO38894.1) was identified in different Baltic metagenomes (JGI Genomes IDs: 3300076127, 3300077758, 3300078340, 3300078341, 3300078342, and NCBI Genome ID: LIAY01000086.1) (S1 Fig). The ArsMB sequence, along with 40 other experimentally characterized bacterial PKS III sequences (S2 Table), was subjected to multiple alignments using Clustal Omega with default parameters [43], via the European Bioinformatics Institute (EMBL-EBI) [44**]**. Manual review of the multiple alignments was performed to identify conserved catalytic residues from PKSs III [36,38]. The putative PKS III ArsMB was then selected for further heterologous expression and bioactivity assays. The biosynthetic gene cluster encoding *ars*MB was analyzed using the online bioinformatics software antiSMASH 7.0 [45]. Functional prediction of proteins encoded by the *ars*MB gene cluster was carried out using the CDD/SPARCLE [40,41] and UniProt (https://www.uniprot.org/) databases. Subcellular localization of proteins was predicted using the CELLO v.2.5 [46].

### Phylogenetic analysis of PKS III

The phylogenetic tree was constructed from an alignment of 78 amino acid sequences from several characterized PKSs III, including 40 bacterial, 12 fungal, 18 plant, 4 protist, and 1 from uncultured microorganisms. Three hypothetical PKSs III-like sequences were used to improve the fit of some clades (S3 Table). The sequences of nine β-ketoacyl-acyl carrier protein synthases III (FabH) were used as an outgroup. The alignment of all amino acid sequences was performed using the Clustal Omega algorithm with default parameters [43] via the European Bioinformatics Institute (EMBL-EBI) [44]. The evolutionary relationship of PKS III was inferred using the Maximum Likelihood method [47], which performed 1000 bootstrap replicates and selected the best-fit amino acid substitution model (LG + F + R5). The tree was constructed using the IQ-tree web server [48], and the consensus tree was visualized in Interactive Tree of Life (iTOL) v5 (https://itol.embl.de/) [49].

### Structural modeling of ArsMB

AlphaFold2-mutimer structural modeling of ArsMB was performed using the ColabFold v1.5.5 patch server (https://github.com/sokrypton/ColabFold/blob/main/AlphaFold2.ipynb) with the amino acid sequence in homooligomer (1:1) configuration and Amber-based energy minimization (num_relax = 5) settings, along with default parameters [50,51]. The best of five generated models was selected based on the highest predicted Local Distance Difference Test (pLDDT) value. The model was validated using MolProbity [52]. Structural analysis was conducted by manual inspection using UCSF ChimeraX v1.5 [53] and the molecular graphics program CCP4mg v2.10.11 [54]. A prediction of the cavities in ArsMB was performed using the CavityPlus web server [55]. The cavities with the highest DrugScore and those that matched with previous crystallized structures were selected. All graphical representations were made with CCP4mg.

### *In vivo* synthesis of ArsMB-derived polyketides

*E. coli* carrying the IPTG-inducible episomal *lac*::*ars*MB circuit was prepared. The IPTG-inducible *lac*::*ars*MB circuit was constructed by inserting the *E. coli* codon-optimized synthetic cistron *ars*MB, synthesized by Integrated DNA Technologies (Iowa, USA), into the pET-22b(+) vector (Novagen) using *Nde*I and *Not*I sites. The resulting pArsMB was used to transform *E. coli* BL21(DE3)pLysS cells. A single colony of *E. coli* BL21(DE3)pLysS/pArsMB was inoculated into 5 mL of LB medium containing 250 μg mL^−1^ ampicillin and incubated overnight at 37°C. Once it reached log phase, the culture was aliquoted into a sterile solution containing 30% (v/v) glycerol and stored at −80°C. The empty vector pET-22b(+) was used as a negative control. ArsMB-derived polyketides were synthesized by *E. coli*/ArsMB using a modified two-stage culture strategy to produce acetyl-CoA-dependent metabolites in a glucose M9 mineral medium (per liter: 6 g Na_2_HPO_4_; 3 g KH_2_PO_4_; 0.5 g NaCl; 1 g NH_4_Cl; 2 mM MgSO_4_; 0.1 mM CaCl_2_; and 0.01 g Vitamin B1) [56,57]. Briefly, overnight cultures of cells grown in LB medium with 250 μg mL^-1^ ampicillin (37 °C, 200 rev min^-1^) were used to inoculate 250 mL of glucose-supplemented (5 g L^-1^) M9 minimal medium at an initial optical density at 600 nm (OD_600nm_) of 0.1. Upon reaching an OD_600nm_ of 0.6, cells were harvested by centrifugation (4000 rev min^-1^) and resuspended in 1 L of fresh glucose-supplemented (5 g L^-1^) M9 medium with 250 μg mL^-1^ ampicillin to a starting OD_600nm_ of 0.1. When the culture reached an OD_600nm_ of 0.6, expression was induced with 10 μM IPTG, and ArsMB-derived polyketide production proceeded at 30°C and 200 rev min^-1^ for 12 h. The biomass was harvested by centrifugation (4000 rev min^-1^, 20 min, 4°C) to separate it from the culture medium at the end of production. Both biomass and culture medium were reserved for qualitative product analysis.

### Qualitative product analysis

The products of both biomass and culture medium were analyzed. The biomass was resuspended in distilled water (0.5 mL g^-1^ wet biomass) and sonicated on ice for 30 min at 25−29% amplitude. The sonicated biomass suspension and the culture medium were extracted three times with 1.5 volumes of ethyl acetate (EtOAc) per volume of sample. The organic phase of each EtOAc extract was recovered and concentrated under reduced pressure in a rotary evaporator (240 mbar, 66°C). Both concentrated crude extracts from biomass and culture medium were subsequently evaluated by qualitative thin-layer chromatography (TLC). Qualitative TLC alkylresorcinol analysis was performed using a Fast Blue B specific staining method for phenolic lipids [58] with some modifications. Briefly, the samples were separated on reverse-phase silica gel 60 RP-18 F_254_S, developed with CHCl_3_/MeOH (30:70, v/v) and revealed with Fast Blue B reagent (No. Cat. D9805; Sigma-Aldrich; 5 g L^-1^ in 10% acetic acid) using resorcinol as reference standard (No. Cat. 398047; Sigma-Aldrich).

### Extraction and purification of cell-associated ArsMB-derived alkylresorcinols

The same two-stage cultivation strategy was used for 30 L scaled-up (pilot-scale) to produce ArsMB-derived alkylresorcinol in glucose M9 mineral medium. Briefly, a overnight culture of cells grown in LB medium with 250 μg mL^-1^ ampicillin (37 °C, 200 rev min^-1^) were used to inoculate 5 L of glucose-supplemented (5 g L^-1^) M9 minimal medium at an initial optical density at 600 nm (OD_600nm_) of 0.1. Upon reaching an OD_600nm_ of 0.6, cells were harvested by centrifugation (4000 rev min^-1^) and resuspended in 30 L of fresh glucose-supplemented (5 g L^-1^) M9 medium with 250 μg mL^-1^ ampicillin to a starting OD_600nm_ of 0.1 (30°C, 150 rev min^-1^, 1 vvm). When the culture reached an OD_600nm_ of 0.6, expression was induced with 10 μM IPTG, and ArsMB-derived alkylresorcinol production proceeded for 8 h (30°C, 150 rev min^-1^, 1 vvm). To isolate cell-associated alkylresorcinols, the biomass was extracted three times with ethyl acetate (1.5 volumes) and evaporated to dryness under reduced pressure. The methanol-soluble portion (56 mg) from the crude extract (100 mg) was loaded onto a Sephadex LH-20 column (13 cm x 2 cm I.D.; Merck KGaA, Darmstadt, Germany) and eluted with methanol, yielding 18 individual 2-mL fractions. GC-MS analysis was performed on the Fast Blue B-positive fraction 14 (1.1 mg).

### GC-MS analysis

Gas Chromatography-Mass Spectrometry (GC-MS) of Fast Blue B-positive fraction 14 was performed on an Agilent Technologies 7890B chromatograph equipped with a HP-5MS capillary column (30 m x 0.25 mm x 0.25 μm, with 5% methyl phenyl siloxane) and a single quadrupole mass spectrometer (Agilent Technologies 5977A). The injector temperature in split 15:1 mode was 280 °C. The temperature program started at 100 °C for 1 min, then increased at 8 °C/min to 310 °C. Mass spectra were recorded using electron impact ionization at 70 eV electron energy over the range 30–600 m/z. The results were acquired and processed with Mass Hunter Qualitative Analysis B.07.00 software. Identification of components was carried out by comparing the experimental mass spectrum with the NIST 14 reference library, accepting matches >80% probability. Retention indices are reported as linear retention indices (LRI, van den Dool–Kratz): LRI = 100n + 100(t_Rx_ − t_Rn_)/(t_RN_ − t_Rn_), where t_Rx_ is the retention time of the analyte and t_Rn_ and t_RN_ are those of the n-alkanes eluting immediately before and after it [59]. A homologous n-alkane series was run under identical chromatographic conditions. Compounds were assigned by comparison of their experimental mass spectra with the NIST 14 reference library together with their LRI. Matches ≥87% were required for the compounds reported in Table 1; the complete peak list for the fraction, including lower-scoring assignments, is given in **S4 Table**.

**Table 1:** Compounds identified by GC-MS in *E. coli*/ArsMB biomass extract.

Compound	*t*_R_ (min)	LRI exp	ΔLRI	Formula	Calculated monoisotopic mass	ions (*m/z*)	Area (%)	µg in F14	NIST match (%)
**ars1** 5-pentadecyl-1,3-benzenediol	24.33	2803.5	+12.5 (+0.45%)	C_21_H_36_O_2_	320.2715	320 [M]⁺•, 166, 137, **124**, 123	1.060	11.7	87.1
**ars2** 5-tridecyl-1,3-benzenediol	22.42	2592.7	+1.7 (+0.07%)	C_19_H_32_O_2_	292.2402	292 [M]⁺•, 151, 137, **124**, 123	0.035	0.38	90.3
**Palmitic acid** C16:0	15.79	—	—	C_16_H_32_O_2_	256.2402	256 [M]⁺•, 213, 185, 129, **73**, 60	36.887	405.8	98.3

Base peak in bold. LRI, linear retention index (van den Dool–Kratz), determined only for the alkylresorcinols. ΔLRI, deviation from the value expected for the 5-*n*-alkylresorcinol homologous series (2591 for C13; 2791 for C15); ΔLRI(ars1 − ars2) = 210.8 units, equivalent to two methylene increments. Amounts in methanolic fraction 14 were estimated from total ion chromatogram peak areas relative to the 1.1 mg of fraction recovered. The complete peak list of the fraction is given in S4 Table.

### Effect of H_2_O_2_ exposure on *E. coli*/*ars*MB

An overnight M9 culture of *E. coli* BL21(DE3)pLysS/pArsMB, hereafter referred to as *E. coli*/arsMB, was used to inoculate 5 mL of M9 medium with 5 g L^-1^ glucose and 200 μg mL^−1^ ampicillin at an initial OD_600nm_ of 0.1 and incubated at 37°C and 250 rev min^-1^. After induction by adding a final concentration of 10 μM IPTG to the medium (OD_600nm_ of approximately 0.6), the temperature was lowered to 30°C, and the cells were grown for 6 h. The culture was then diluted to an OD_600nm_ of 0.05 in a 96-well microplate (ThermoScientific™ Nunc™) containing M9 medium with 5 g L^-1^ glucose, 200 μg mL^−1^ ampicillin, and 10 μM IPTG. H_2_O_2_ (10 μL) was added from a 3% (w/v) solution (Jaloma), freshly diluted in sterile Milli-Q water, to a final concentration of 0.2–1 mM in a total volume of 150 μL per well. Control culture received the same volume of water as H_2_O_2_. The assay was performed at 30°C with continuous shaking. Growth kinetics were determined by measuring OD at 600 nm every 7 min for 16 h using an automated microplate reader (ELx808, BioTek). Specific growth rates (μ) were calculated by determining the slope of the log-linear region using a linear regression (μ = h^-1^). The *E. coli* carrying the empty pET-22b(+) was used as a negative control.

### Muralytic activity assessment with endolysin

The lytic activity assessment was performed by using three endolysins: KZ144 (NCBI ID: NP_8N03710; [60], LysPA26 (NCBI ID: ARB16052.1; [61], and in-house characterized OsLys (NCBI ID: ASV42463.1) [62]. Muralytic activity was determined by turbidimetric methods, using the chloroform-sensitized cells method to expose the PG substrate, as described previously [62], in a sterile, flat-bottom 96-well microplate (Thermo Scientific™ Nunc™). First, the outer membrane was removed to expose the peptidoglycan (PG) as follows: the bacterial cells were permeabilized with chloroform, a standard procedure to evaluate the bactericidal activity of endolysin. Independently, an inoculum (0.1 OD_600nm_) in 100 mL of TB medium (per liter: 20g tryptone, 24 g yeast extract, 5 g glycerol, 0.017 M KH_2_PO_4_ and 0.072 M K_2_HPO_4_), with 200 μg mL^-1^ ampicillin, was prepared from a pre-inoculum of *E. coli*/pET22b and *E. coli*/arsMB and incubated at 37°C with orbital shaking (150 rev min^-1^) for approximately 2.5 h until reaching an OD_600nm_ of 0.6. ArsMB synthesis was induced with 10 μM IPTG, followed by incubation at 30°C with orbital shaking (150 rev min^-1^) for 6 h (OD_600nm_ approximately 2). The biomass was harvested by centrifugation at 4,000 rev min^−1^ for 20 min, and the supernatant was discarded. The chloroform treatment involved incubating the cellular pellet in 120 mL of chloroform-saturated 20 mM sodium phosphate buffer, pH 7.4, at 50 rev min^-1^ for 1 h. The sensitized cellular pellet with chloroform was harvested and washed with 120 mL of distilled water. The turbidity-reduction assay was conducted using a suspension of sensitized cells (OD_600_ = 0.6) in buffer containing 50 mM Tris-HCl and 100 mM NaCl, pH 7.0, with the OD600nm adjusted to 1.2. The reaction mixture (210 μL) consisted of 180 μL of sensitized *E. coli*/arsMB suspensions with a final OD_600nm_ of approximately 1, along with 30 μL of endolysin solution at final concentrations of: KZ144 0.75, 5.0, and 7.5 μg mL^-1^; LysPA26 0.75, 7.5, and 15.5 μg mL^-1^; OsLys 0.75, 7.5, and 12.5 μg mL^-1^. Sensitized *E. coli* cells carrying empty pET22b (*E. coli*/pET22b) were used as a control. OD_600nm_ measurements were performed in triplicate at 30°C using a MultiSkan microplate reader (Thermo Scientific™) for 12 min, with readings taken every 30 s and 5 s after shaking. One unit of specific muralytic activity is defined as the decrease in ΔOD_600_ per minute per μg of endolysin (U/μg) [63].

### Statistical analysis

Each replicate contributed a single independent observation as each kinetic trace was reduced to summary parameters computed separately for every replicate. For the H_2_O_2_ assay, every well was summarised by three parameters: the area under the growth curve between 0 and 16 h (AUC, ΔOD_600_·h), the biomass gain at 16 h (ΔOD_600_,16h), and the recovery lag λ, defined as the time required to reach ΔOD_600_ = 0.05. For the endolysin assay, the normalised turbidity of every well was fitted individually to a one-phase exponential decay, ΔOD_600_(*t*)/ΔOD_600_(0) = *P* + (1 − *P*)e^−kt^, where k (min^−1^) is the first-order lysis constant and *P* the residual turbidity plateau; the initial lysis rate v0 was obtained as the slope over the first 2 min, and the lysis AUC was integrated between 0 and 12 min. Per-well parameters were analysed by two-way ANOVA (strain × concentration) with partial η² as the effect-size measure. Strain contrasts at each concentration were evaluated by Welch’s t-test and corrected for multiple comparisons with the Holm–Bonferroni procedure across the whole family of concentrations. Effect sizes are reported as the difference between means with its 95% confidence interval and as Hedges’ g, alongside the p values. As a sensitivity analysis, the complete time courses were modelled by LMM (ΔOD_600_ ~ strain × time × concentration) with a random intercept and a random slope per well, which accounts explicitly for the repeated-measures structure of the data. Analyses were performed in Python 3.11 (SciPy, statsmodels 0.15) and GraphPad Prism v8.0.1 (San Diego, California, USA), with α = 0.05.

## Results and discussion

### Identification of an emerging PKS III-encoding gene from marine bacteria

To identify putative PKS III genes involved in the biosynthesis of alkylresorcinols in marine bacteria, a database-mining approach was applied to approximately 390 publicly available metagenomic datasets from samples collected across different locations in the Baltic Sea. A putative PKS III-encoding gene named *ars*MB (NCBI ID: KRO38894.1) was detected in several scaffold genomes of uncultured *Pontimonas* sp from different samples of microbial communities living on the surface of the Baltic Sea near Öland, Sweden (56°55’51.1"N 17°03’38.4” E) (JGI Genomes IDs: 3300076127, 3300077758, 3300078340, 3300078341, 3300078342, and NCBI Genome ID: LIAY01000086.1, [64]). Sequence analysis reveals that ArsMB consists of a mature protein of 349 amino acid residues and shares < 50% sequence identity (100% query coverage) with 41 characterized bacterial PKSs III (**S2 Table**), including the presence of the highly conserved catalytic triad Cys139-His275-Asn308 involved in the priming and extension of a polyketide chain (Fig 1A) [65]. Although no PKS III has been characterized from the genus *Pontimonas*, members of the class Actinomycetes are well-established as prolific producers of polyketides [66,67]. Indeed, ArsMB shares 55–40% sequence identity (100% coverage) with putative PKSs III from different species of Actinobacteria found in the Baltic Sea, such as *Aquiluna* sp (JGI ID: Ga0665900_0005460_7472_8527), unclassified Microbacteriaceae (NCBI ID: KRO48341.1), *Mycobacterium* sp (JGI ID: Ga0210003_10034323), *Streptomyces* sp (JGI ID: Ga0665898_0096702_704_1777), *Nocardioides* sp (Ga0634394_22598_1499_2557), among others.

**Fig 1:**
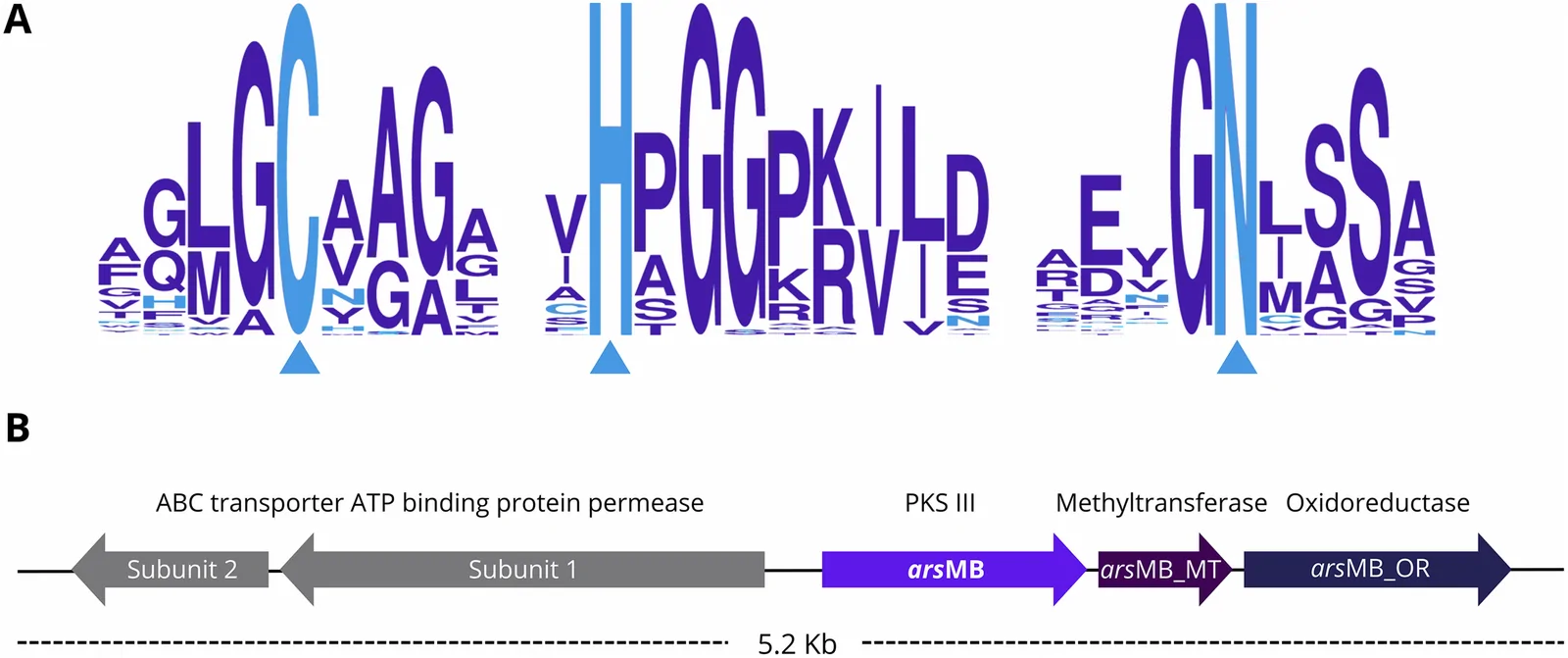
Identification of a putative PKS III of marine origin. (**A**) Multiple amino acid sequence alignment of characterized PKSs III and ArsMB, showing conserved motifs. The blue arrowheads indicate the highly conserved catalytic triad Cys-His-Asn of PKSIII. Graphic created on WebLogo.berkeley.edu. (**B**) Genetic organization of the predicted *ars*MB biosynthetic gene cluster (5239 bp). The genes encoding the *srs*ABC-like cassette: PKS III *ars*MB, methyltransferase *ars*MB_MT, and oxidoreductase *ars*MB_OR are shown. The two subunits of a putative ABC transporter ATP-binding protein permease are also shown. Arrows indicate the position and direction of the open reading frames.

The approximately 5.2 Kb gene cluster containing *ars*MB (NCBI ID: LIAY01000086.1) comprises five open reading frames (ORFs). The *ars*MB biosynthetic gene cluster encodes a classical *srs*ABC-like operon dedicated to producing amphipathic membrane-active ARs [25] (Fig 1B). Accordingly, the *ars*MB gene codifies the core biosynthetic enzyme, consisting of a PKS III chalcone/stilbene synthase sharing 48% sequence identity (100% query coverage) with the alkylresorcinol synthase, SrsA (NCBI ID: BAG17301.1) (Fig 1B). The *ars*MB ORF is followed by two putative tailoring enzymes of the alkylresorcinol scaffold: O-methyltransferase, ArsMB_MT (NCBI ID: KRO38895.1), and a flavoprotein hydroxylase, ArsMB_OR (NCBI ID: KRO38896.1). The *ars*MB_MT gene codifies for a putative membrane-bound enzyme that includes an isoprenylcysteine carboxyl methyltransferase (ICMT) domain that shares 52% average sequence identity (100% query coverage) with the O-methyltransferase SrsB (NCBI ID: BAG17300.1). Furthermore, *ars*MB_OR encodes a putative cytoplasmic enzyme with an NAD(P)/FAD-dependent oxidoreductase domain and contains a sequence motif found in flavoprotein hydroxylases (Fig 1B) [68]. This hypothetical protein shares 43% average sequence identity (100% query coverage) with the FAD-dependent hydroxylase SrsC. Altogether, the BGC analyzed by AntiSMASH showed 66% gene similarity to the alkylresorcinol-producing operon *srs*ABC from *S. griseus* [25]. The *srs*ABC-like and *srs*AB-like operons are functionally active with the type III PKS SrsA catalyzing the synthesis of phenolic lipids, alkylresorcinols, and alkylpyrones, while SrsB and SrsC produce methylated and hydroxylated derivatives, respectively [25]. Notably, these phenolic lipids appear to play a crucial role in maintaining cell wall/membrane integrity and in resistance to β-lactam antibiotics [25]. Earlier studies also reported antimicrobial activity against clinically relevant strains, including *Staphylococcus aureus* and *Aspergillus fumigatus* [69]. In *Mycobacterium*, two polyketide synthase proteins responsible for producing methylated polyketides are essential for mycobacterial survival within biofilms [26]. More recently, a highly conserved BGC producing long-chain alkylpyrones was identified in *Dendrosporobacter quercicolus*, yet is notably absent in related non-sporulating Negativicutes, suggesting a potential physiological role linked to sporulation processes [70]. On the other hand, the *ars*MB *srs*ABC-like operon is preceded by two additional ORFs, annotated as ABC transporter ATP-binding protein permease subunit 1 and 2, and located 219 and 2102 bp upstream of *arsMB*, respectively (Fig 1B). Although ABC transporters are frequently associated with detoxification or the export of endogenous secondary metabolites [71], the roles of the two putative ABC transporter permeases linked to the *ars*MB BGC core remain unclear.

### Phylogenetic analysis of ArsMB

Phylogenetic analysis was conducted to determine the evolutionary relationships of ArsMB, including PKS III reference sequences from plants, fungi, and protists, and nine KAS III (FabH) sequences as an outgroup (Fig 2). The resulting tree revealed that bacterial PKS III enzymes cluster into three distinct clades, clearly separated from their fungal and plant counterparts (bootstrap values > 70%), consistent with previous findings [21,72]. Bacterial clades are designated by the letter B, with the accompanying number indicating the compound they produce (Fig 2). Clade B1 is further subdivided into subclades B1-1 and B1-2. The B1-1 subclade consists of PKS III enzymes capable of utilizing long-chain acyl-CoA/ACP starter units, as well as malonyl-CoA (MCoA) and methyl malonyl-CoA (MMCoA) as extenders in two to three condensation steps, leading to the production of methylated alkylpyrones and alkylresorcinols (Fig 2). In contrast, the B1-2 subclade favors short- and medium-chain acyl-CoA/ACP starter units, along with MCoA, MMCoA, or ethyl malonyl-CoA (EMCoA), resulting in a diverse array of pyrones, including germicidins, alkylhydroxypyrones, violapyrones, and presulficidins, as well as phloroglucinol and tetrahydroxynaphthalene (THNS) (Fig 2). As in the case of the GcS, RppA, and PhlD enzymes of *S. coelicolor* A3(2), *S. griseus* and *Pseudomonas protegens* Pf-5, respectively [37,73,74]. Clade B2 exclusively comprises DpgA enzymes, which are involved in the biosynthesis of vancomycin-type antibiotics [75–77]. These enzymes catalyze the condensation of four MCoA molecules to produce 3,5-dihydroxyphenylacetyl-CoA (DpA-CoA) [78,79]. Clade B3 is the most heterogeneous, encompassing both bacterial and eukaryotic PKS III enzymes. This clade is subdivided into B3-1 and B3-2 subclades. The B3-1 utilizes long-chain acyl-CoA/ACP starters with MCoA as an extender to produce alkylresorcinols [80–82]. Subclade B3-2 integrates the bacterial MtbPKS18 alongside eukaryotic PKSs III from brown algae and slime mold [72]. Both MtbPKS18 and SbPKS utilize long-chain acyl-CoA/ACP starters with MCoA to produce alkylpyrones, while the remaining eukaryotic PKSs III produce acylphloroglucinols [72,83]. Phylogenetic clustering of PKS III enzymes reveals a putative correlation with catalytic function, with distinct clades grouping synthases responsible for structurally related metabolites (Fig 2). ArsMB was clustered in subclade B1-1 (bootstrap value of 99%) among PKSs III enzymes involved in phenolic lipid biosynthesis, including alkylpyrones, alkylresorcinol, and alkylresorcilic acid synthases. Despite the complex evolution of PKS III sequences, which may reflect both speciation and differences in reaction type, this result suggests that ArsMB is a hypothetical phenolic lipid synthase with the potential to condense MCoA- and MMCoA units as extender substrates selectively.

**Fig 2:**
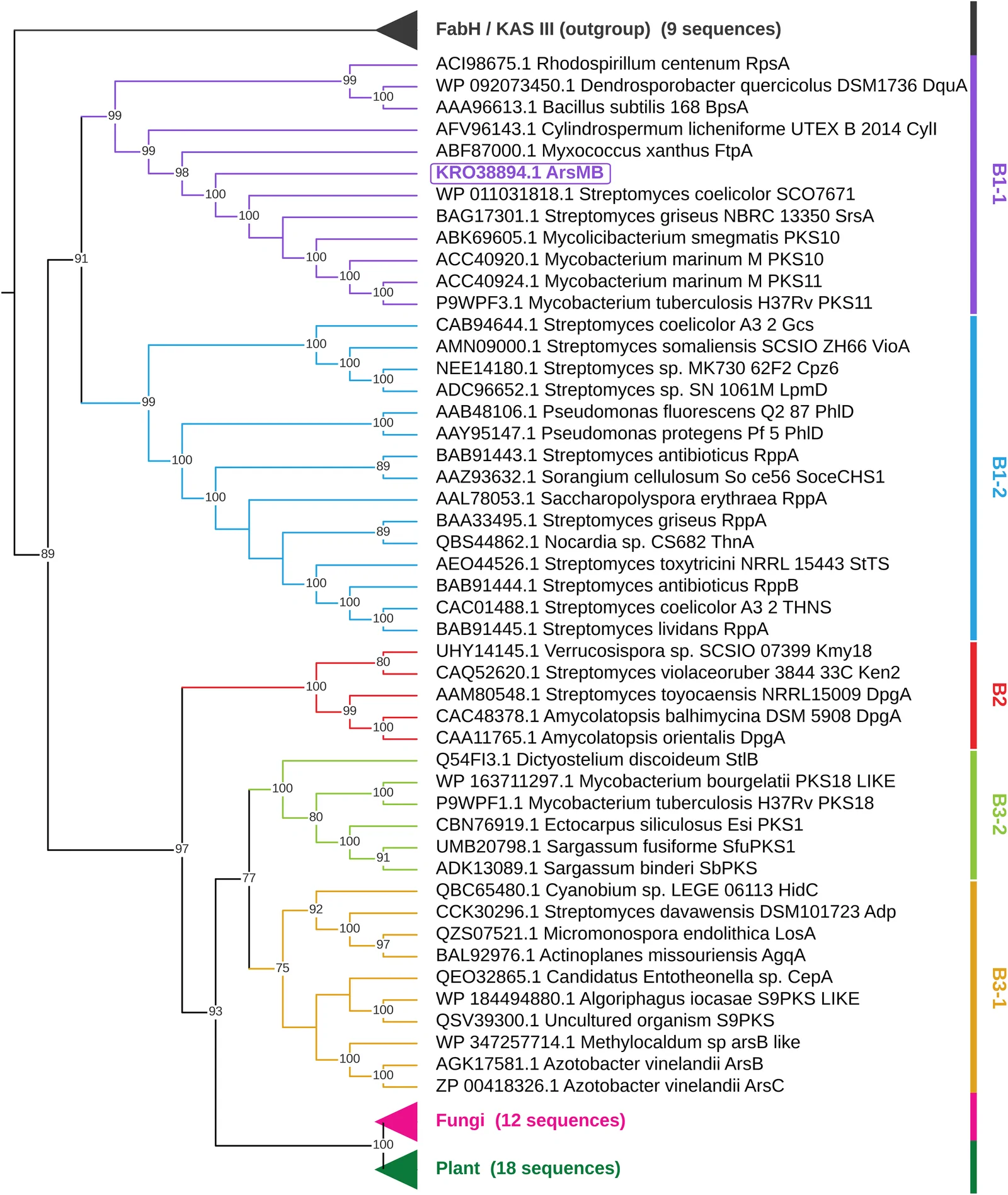
Phylogenetic analysis of ArsMB. Evolutionary relationships were determined by the Maximum Likelihood method based on the LG + F + R5 model using the amino acid sequences of 78 PKSs III. This analysis includes 74 characterized PKSs III from bacterial, fungal, plant, and other eukaryotic origins. Bacterial PKS III sequences include those capable of utilizing long-chain acyl-CoA/ACP (B1-1, purple) to produce alkylpyrones and alkylresorcinols, and those that employ short-chain initiators to produce pyrones (B1-2, blue), as well as those involved in antibiotic biosynthesis (B2, red). Clade B3 grouped PKSs III from bacteria and eukaryotes that produce alkylresorcinols (B3-1, yellow) and alkylpyrones (B3-2, lemon green). ArsMB, highlighted within a purple box, clusters within clade B1-1. Nine *β-*ketoacyl-acyl carrier protein synthase III (FabH) sequences were used as an outgroup (black). The outgroup and the fungal (pink) and plant (green) clades, which were clearly separated from the bacterial PKS III clades, are shown collapsed as triangles with the number of sequences indicated. All sequences are in **S3 Table**. Bootstrap support values, calculated from 1000 iterations, are indicated for each node. Only bootstrap values above 70% were shown. The tree was drawn using Biopython and Matplotlib.

### Structure modeling of ArsMB

The structural architecture (3D structure) of ArsMB was predicted by computational modeling using AlphaFold2-multimer, a deep learning system optimized for protein complexes (Fig 3). The resulting model, with a confidence score of pLDDT > 97%, predicts that ArsMB adopts a homodimeric assembly comprising two structural domains. Each domain exhibits a highly conserved PKS III αβαβα fold, as previously described [84,85], and contains two functionally independent active sites (Fig 3A). The internal active-site cavity contains the catalytic triad Cys139/His275/Asn308, which is required for the priming and extension of polyketides. Additionally, it includes a structurally conserved Pro337 residue located in a β-harpin turn, thought to enhance rigidity and facilitate cyclization (Fig 3B) [70,86]. The predicted ArsMB model identifies two channels converging within the active site cavity: a surface-accessible CoA-binding tunnel and a long, deep hydrophobic substrate-binding tunnel, with Phe189 and Trp228 positioned at its entrance (Figs 3A and 3B). This extensive hydrophobic tunnel resembles those observed in other PKS III enzymes with resolved structures that accommodate long-chain fatty acyl substrates [26,38,79,87,88]. Structural comparison of ArsMB with the alkylpyrone synthase PKS11 from *M.* *tuberculosis* (PDB ID: 4JAO) [38], which superposes 322 Cα with ArsMB with an RMSD of 0.79 Å, allowed the identification of the residues involved in the hydrophobic tunnel cavity. This cavity is predominantly lined with hydrophobic residues, including Val107, Leu237, Cys169, Thr172, Thr186, Ile173, Tyr60, Leu25, Leu53, Arg51, Ile21, and Ile57 (Figs 3C and 3D). Notably, no steric hindrances were observed with the bound palmitate (C16) alkyl chain (Fig 3C). In contrast, superposition with ORAS (265 Cα atoms aligned, RMSD of 1.057 Å, PDB ID: 3EUO) reveals that the side chain of Tyr60 in ArsMB, located in a loop shortened by four amino acids, imposes steric impedance on eicosanoic acid (C20) (Fig 3D). In ArsMB, the acyl-binding tunnel is straight and extends toward the surface rather than inward into the protein, as observed in ORAS [87].

**Fig 3:**
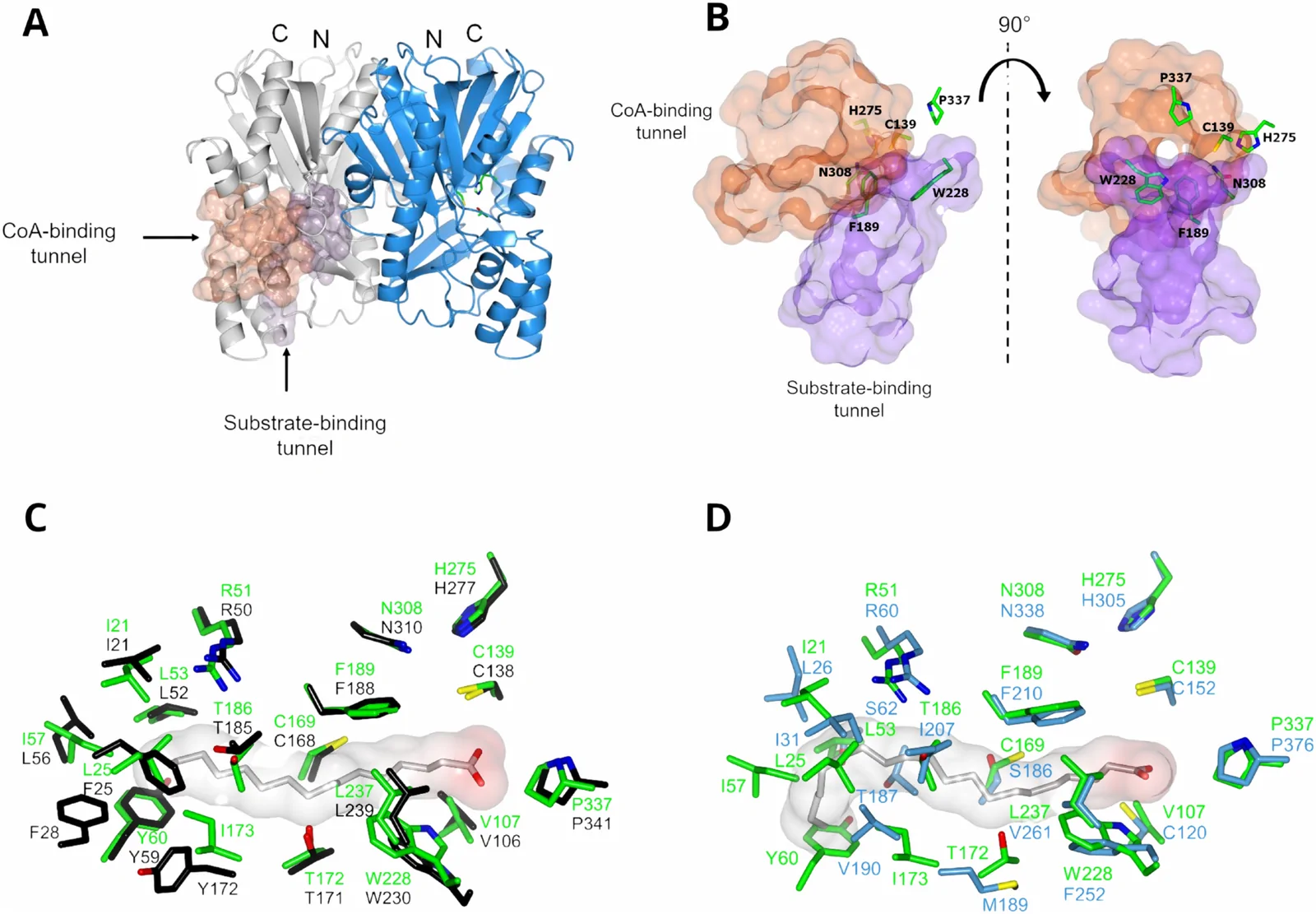
Structural model of ArsMB. (**A**) Homodimeric structure of ArsMB showing the CoA-binding tunnel and the substrate-binding tunnel. Each monomer exhibits an αβαβα fold. (**B**) Surface representation of the acyl-CoA binding tunnel (coral) and the substrate binding tunnel (purple) in ArsMB. Catalytic residues C139, H275, and N308 are shown in green. (**C**) Superposition of the amino acids conforming to the substrate binding tunnel and the catalytic triad in ArsMB (green) and PKS11 (black; PDB ID: 4JAO). (**D**) Superposition of the amino acids conforming the substrate binding tunnel and the catalytic triad in ArsMB (green) and ORAS (blue; PDB ID: 3EUT).

As in other microbial resorcinol/resorcylic acid synthases, the ArsMB model also shows an aromatic residue, Trp228, at the entrance of the substrate tunnel, which has been proposed to play a key role in facilitating cyclization via aldol condensation [88]. According to structural comparisons, the aromatic residue corresponds to Phe252 in ORAS and Trp284 in ArsC (engineered resorcinol synthase; PDB ID: 3VS9) [87,88]. Moreover, some bacterial PKSs III have been reported to accept acyl-ACP starter substrate [88–92]. A cationic residue (Arg/Lys) adjacent to the catalytic triad active site has been shown to be crucial for priming the ACP-bound substrate [36,84]. In ArsMB, this residue corresponds to Arg280 (**S5 Fig**). Overall, the model is compatible with incorporating long-chain fatty acyl-CoA/ACP starter units. The model alone cannot infer the cyclization chemistry.

### Synthesis *in vivo* of the ArsMB-derived 5-n-alkylresorcinol compounds

The *E. coli* metabolic chassis, which carries the IPTG-inducible episomal *lac*::*ars*MB circuit, was used to synthesize ArsMB-derived polyketides. The *in vivo* synthesis was achieved using the classical two-stage production strategy in a glycolytic mineral medium that enhances the acetyl-CoA pool [56,57]. Qualitative TLC of the concentrated ethyl acetate extracts from biomass showed a distinct Fast Blue B–positive resorcinol spot produced by *E. coli*/ArsMB which was absent from the empty-vector *E. coli*/pET22b control (**S6 Fig**), confirming that *E. coli* does not synthesize these compounds natively [78]. No Fast Blue B–positive resorcinol spot was detected in the concentrated ethyl acetate extracts from culture medium, indicating that the products remain cell-associated rather than secreted under these conditions (**S7 Fig**).

Scale-up followed by ethyl acetate extraction gave 100 mg of crude extract from biomass, of which the methanol-soluble portion (56 mg) was fractionated on Sephadex LH-20. GC-MS analysis of the Fast Blue B–positive fraction 14 (1.1 mg) resolved two compounds consistent with 5-n-alkylresorcinols, eluting at 22.42 min (ars2) and 24.33 min (ars1) (Fig 4A; **Table 1**). Both mass spectra share a base peak at *m*/*z* 124 together with ions at *m*/*z* 123 and 137 (Figs 4B and 4C). The ion at **m**/*z* 124 corresponds to the dihydroxytropylium-type fragment diagnostic of a 1,3-dihydroxylated benzene ring bearing an alkyl substituent, and its dominance in both spectra places the resorcinol core in each molecule. Molecular ions were observed at *m*/*z* 320 for ars1 and *m*/*z* 292 for ars2, matching the calculated monoisotopic masses of C_21_H_36_O_2_ (320.2715) and C_19_H_32_O_2_ (292.2402); the corresponding M + 1 isotope abundances, 24% and 21% of the molecular ion, are those expected for 21 and 19 carbon atoms, respectively. On this basis, ars1 and ars2 are assigned as 5-pentadecyl-1,3-benzenediol (adipostatin A) and 5-tridecyl-1,3-benzenediol (grevillol). Retention behavior supports these assignments. The LRI measured for ars2 (2592.7) matches the reference value for grevillol (2591) to within 0.07%. More informatively, the difference between the two compounds, ΔLRI = 210.8, corresponds to two methylene increments (approximately 100 index units each on a non-polar stationary phase), establishing that ars1 and ars2 differ by exactly C_2_H_4_ and belong to the same 5-n-alkylresorcinol homologous series. The LRI measured for ars1 (2803.5) agrees to within 0.45% with the expected value for the C15 homologue on that basis (2791). ars1 was the dominant product, with a peak area ~30-fold that of ars2 (1.060% and 0.035% of the total ion chromatogram; Table 1). Relating these areas to the mass of fraction 14 gives approximately 11.7 µg of ars1 and 0.38 µg of ars2, or ~12 µg total, which is 0.012% (w/w) of the 100 mg crude extract. Similarly low yields were found in the heterologous host, highlighting that overproduction of phenolic lipids alters stability and membrane fluidity, which are detrimental to cell viability [23,93]. The remaining constituents of the fraction are other membrane lipids of the host (**S4 Table**). Palmitic acid (C16:0) was by far the most abundant, accounting for 36.9% of the total ion chromatogram, and its spectrum displayed the m/z 60 and 73 rearrangement ions characteristic of non-esterified carboxylic acids (**Table 1**).

**Fig 4:**
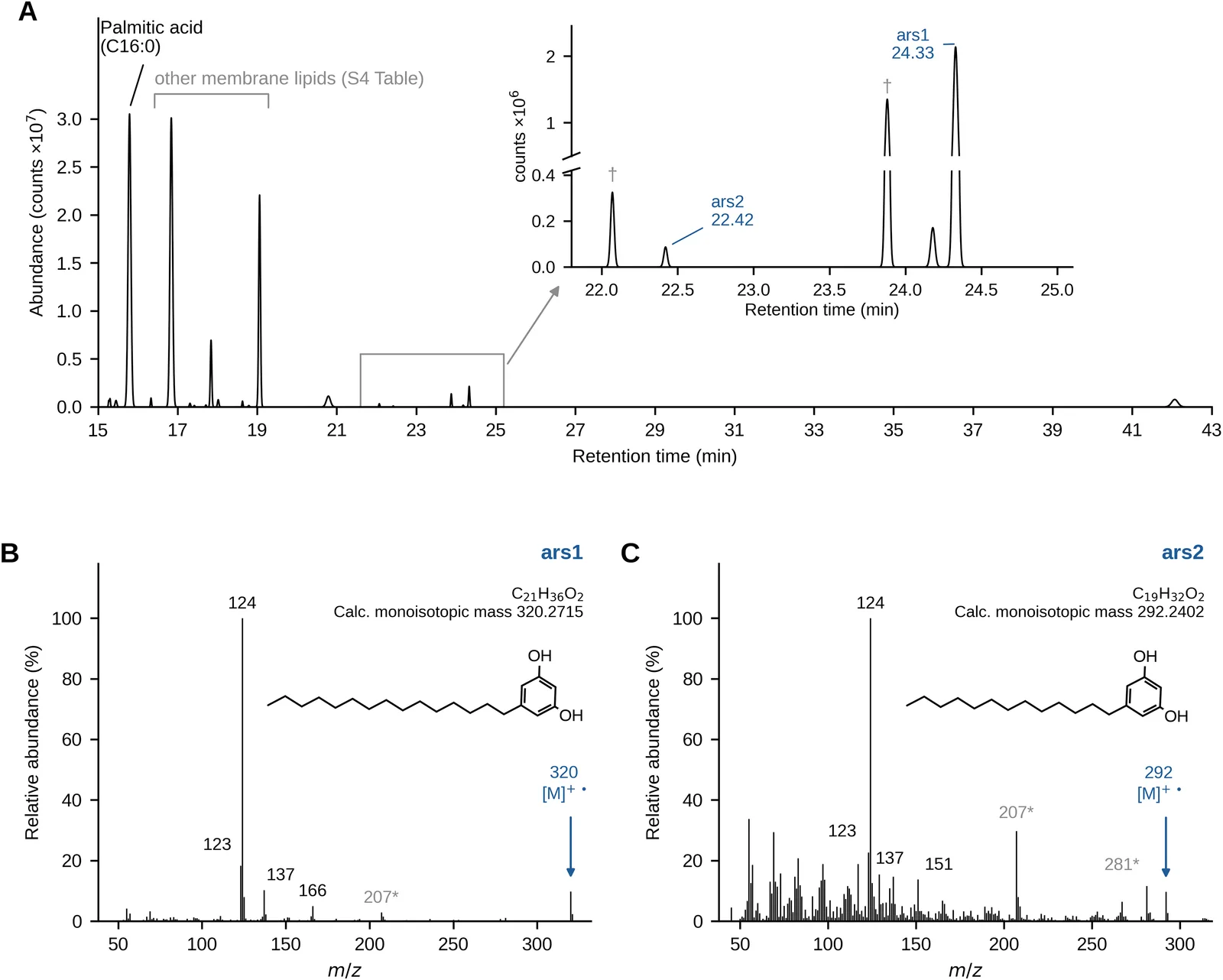
GC-MS profile of the methanolic fraction 14 from *E. coli*/ArsMB biomass extract. (**A**) Total ion chromatogram. Palmitic acid (C16:0), the dominant free fatty acid, is indicated; the remaining membrane lipids are listed in S4 Table. Inset (split ordinate): expansion of the 21.7–25.1 min window showing ars2 (22.42 min) and ars1 (24.33 min). (**B**) Electron-ionization mass spectrum of ars1 and (**C**) of ars2, each shown with the proposed structure and calculated monoisotopic mass; the arrow marks the molecular ion. †, process-derived background (S4 Table); *, polysiloxane column bleed.

Adipostatin A and grevillol are alkylresorcinols isolated from plants and bacterial sources, including *Ginkgo biloba*, *Grevillea robusta*, *Lysimachia japonica*, *Lysimachia tengyuehensis*, and various *Streptomyces* strains [94–97]. These compounds have been investigated for their inhibitory effects on key enzymes involved in metabolic and pathogenic pathways. Applications in which Adipostatin A has been tested include inhibition of glycerol-3-phosphate dehydrogenase (GHDP) [98], contributing to the efficient elimination of the adult-stage *Brugia malayi* parasite [97], and inhibition of the main protease of SARS-CoV-2 [94]. Grevillol, particularly the isolated form from *L. japonica*, has exhibited significant cytotoxicity against various tumor cell lines, including KB, B-16, PC-13, L-5174Y, P-388, and HEp-2, supporting its potential as an antitumor agent [99]. Moreover, ARs from *L. tengyuehensis*, including grevillol, have recently shown inhibitory activity against Methicillin-Resistant *Staphylococcus aureus* (MRSA) and Vancomycin-resistant *Enterococcus* (VRE), highlighting their relevance in combating multidrug-resistant pathogens [100].

### Reconstituting the biosynthetic pathway for ars1 and ars2 in *E. coli* host

Based on metabolomic experimental data, we propose a putative biosynthetic pathway for ars1/ars2. The synthesis of alkylresorcinols ars1 and ars2 in the *E. coli*/arsMB strain relies on the coordination of three main metabolic steps. First, glycolysis utilizes glucose from the M9 mineral medium as the unique carbon source to direct metabolic flux toward acetyl-CoA [56,57]. Second, acetyl-CoA carboxylase converts a portion of this pool into malonyl-CoA, supplying the extender units. Finally, the recombinant ArsMB enzyme condenses malonyl-CoA with endogenous fatty acyl thioester starters (Fig 5). The composition of the fraction from which ars1 and ars2 were isolated supports this route directly. Palmitic acid (C16:0) accounted for 36.9% of the total ion chromatogram and was recovered as a free fatty acid, consistent with release of fatty acyl-ACP by thioesterases and subsequent activation by acyl-CoA synthetase (FadD) (**Table 1**; **S4 Table**; Fig 5). Consistent with structural modeling of the ArsMB active site, the C15 side chain of ars1 derives from a C16 (palmitic acid) starter unit, matching the predominant free fatty acid observed experimentally (**Table 1**; **S4 Table**). Conversely, while ars2 requires a C14 (myristic acid) starter unit, free myristic acid was below the detection limit in this fraction; nevertheless, the 30.5-fold excess of ars1 over ars2 closely mirrors the natural availability of these acyl precursors in the host cell.

**Fig 5:**
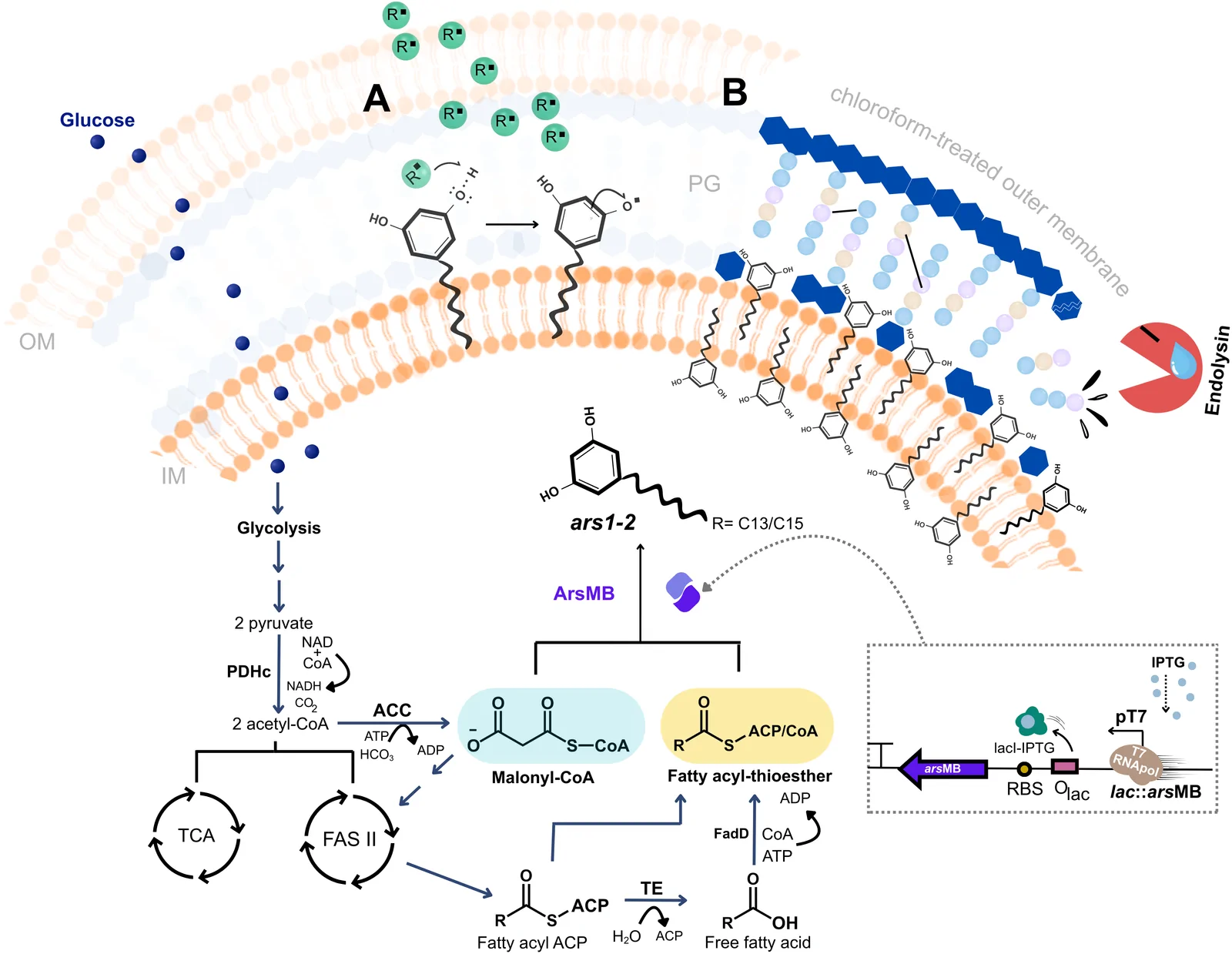
The *in vivo* synthesis strategy to produce host-protective ArsMB-derived alkylresorcinols. The *E. coli* metabolic chassis supplying acyl-CoA and fatty acyl-thioester precursors from glucose is shown. The inducible synthetic genetic circuit *lac*::*ars*MB (pArsMB) is shown in the dashed box (bottom right). A schematic representation of the insertion of amphipathic ars1/ars2 compounds into the inner membrane (IM) is proposed based on literature [101**–**102]. Illustrative models for the hypothetical antioxidant radical scavenging and muralytic activity inhibition effect of the alkylresorcinols ars1 and ars2 covering the inner membrane of *E. coli* carrying *lac*::*ars*MB, are shown in A and B.

### Lytic-resistant tests of the host carrying ars1 and ars2

#### Effect of peroxide resistance.

Alkylresorcinols have been shown to associate with cellular membranes, exerting a protective effect against xenobiotics such as free radicals, largely due to their amphiphilic structure [25,30,103,104]. To evaluate the influence of cell-associated ars1 and ars2 biosynthesis in *E. coli,* an *in vivo* assay using H_2_O_2_ was performed. *E. coli*/*ars*MB (producer strain) and *E. coli* (non-producer control) were cultured in M9 medium and exposed to increasing concentrations of H_2_O_2_ for 16 h. Growth kinetics showed that H_2_O_2_ exposure reduced growth in both strains in a concentration-dependent manner (Fig 6A). To quantify the effect without treating successive readings from the same well as independent observations, we reduced each growth curve to three per-well parameters (AUC0–16h, ΔOD600,16h, and the recovery lag λ; **S9 Table**). Two-way ANOVA on AUC_0–16h_ revealed a significant strain × H_2_O_2_ interaction (*F*_4,18_ = 38.4, *p* < 0.0001, partial η^2^ = 0.89), indicating that the two strains respond differently to peroxide challenge rather than exhibiting a constant difference. In the absence of H_2_O_2,_ the two strains were indistinguishable (AUC_0–16h_ difference −0.14 ΔOD_600_·h, 95% CI −0.43 to 0.15, *p* = 0.42; λ difference 0.14 h, 95% CI −0.18 to 0.45, *p* = 0.20), showing that the metabolic burden of ArsMB expression does not by itself alter growth under these conditions. Under peroxide challenge, the producer strain resists the lytic effect: at 0.2 mM H_2_O_2_ its AUC_0–16h_ exceeded that of the control by 1.64 ΔOD_600_·h (95% CI 1.01 to 2.26; Holm-adjusted *p* = 0.020; Hedges’ *g* = 6.0) and its biomass gain at 16 h was 4.9-fold higher (ΔOD_600_,16h 0.399 ± 0.028 versus 0.082 ± 0.046; difference 0.317, 95% CI 0.223 to 0.412; *p* = 0.005; *g* = 6.6). At 0.4 mM, the control strain did not grow at all, whereas the producer strain still accumulated biomass (ΔOD_600_,16h 0.132 ± 0.008 versus −0.001 ± 0.002; difference 0.133, 95% CI 0.114 to 0.152; *p* = 0.003; *g* = 17.3).

**Fig 6:**
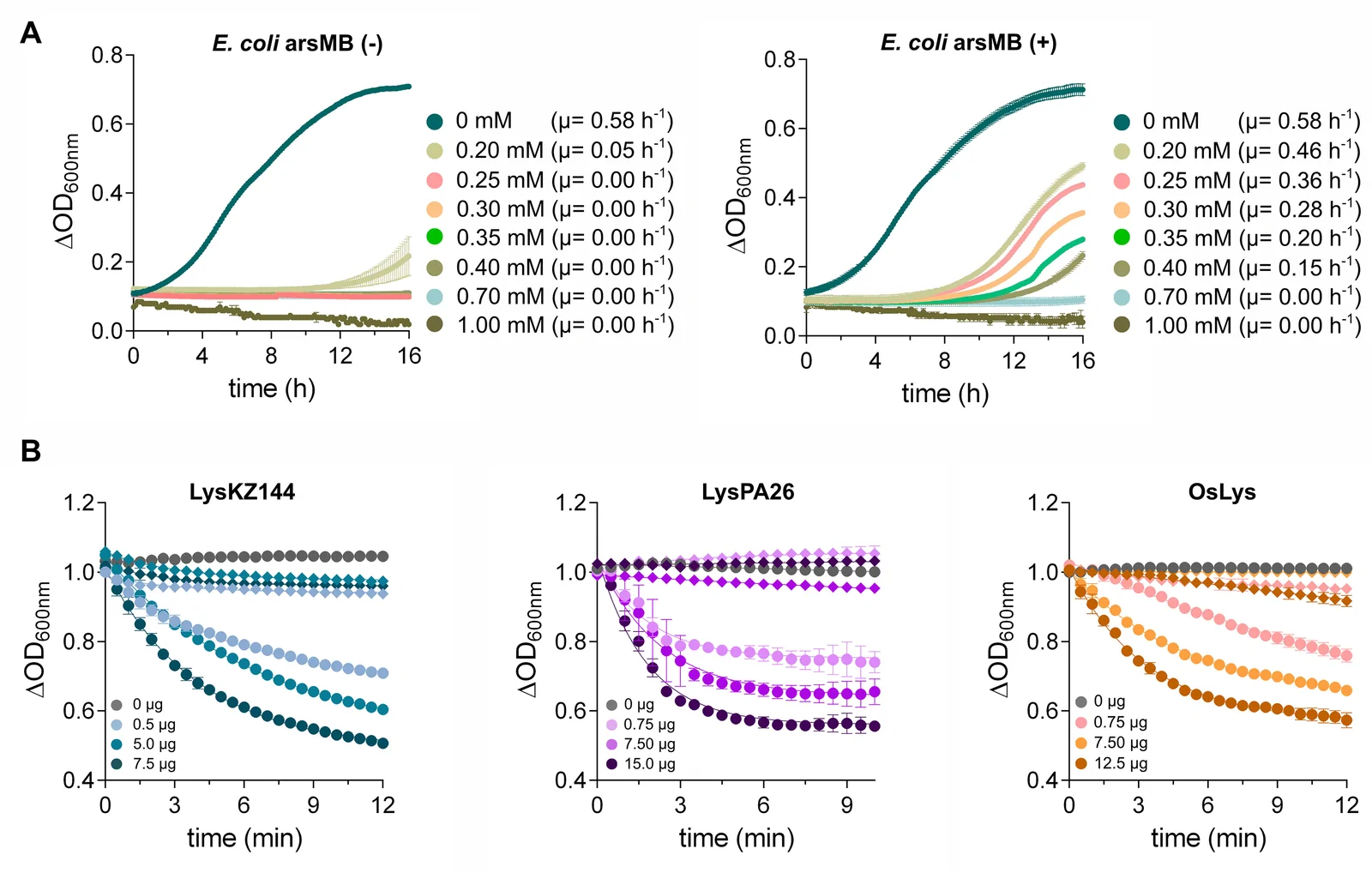
Effect of peroxide exposure and impact of muralytic digestion with endolysins on *E. coli* host. **(A)** Growth kinetics of non-ArsMB-producing *E. coli* (*E. coli* arsMB (-)) and ArsMB-producing *E. coli* (*E. coli* arsMB (+)) exposed to varying concentrations of H_2_O_2_ (0-1 mM) monitored over a 16 h period. Specific growth rates (μ), defined as the slope of the log-linear region (μ = h^-1^), are shown for each peroxide concentration. **(B)** Lysis kinetics graphics of peptidoglycan-exposing *E. coli* arsMB (-) (circles) and *E. coli* arsMB (+) (diamonds) cells treated with different concentrations of endolysins KZ144, LysPA26, and OsLys are illustrated. The negative control is untreated, peptidoglycan-exposing *E. coli* cells (gray circles). Symbols represent the mean of three independent cultures, and error bars represent the standard deviation. Individual replicate traces are shown in S8 Fig, and all per-well derived parameters, together with the corresponding effect sizes, 95% confidence intervals, and Holm-adjusted p-values, are listed in **S9****-****S10 Tables**. Statistical inference was performed on per-well summary parameters (AUC, ΔOD_600_,16h, recovery lag λ, lysis constant *k*) rather than on individual time points, and confirmed by a linear mixed-effects model of the complete time courses.

The protection was expressed primarily as a shortening of the recovery lag rather than as a faster exponential rate: at 0.2 mM H_2_O_2_ the producer strain resumed growth after 8.6 ± 0.7 h versus 15.2 ± 1.2 h for the control (difference −6.6 h, 95% CI −8.9 to −4.3; *p* = 0.002; *g* = −5.6), and at 0.25–0.40 mM only the producer strain recovered within the 16 h of the assay. Consistently, the LMM fitted to the complete time courses at 0.2 and 0.4 mM (1,653 readings from 12 wells, random intercept and slope per well) returned a significant strain × time interaction (*β* = +0.041 ΔOD_600_ h^−1^, 95% CI 0.034 to 0.047, *p* < 0.0001). The effect was nevertheless conditional: at 0.7 and 1.0 mM H_2_O_2_ neither strain grew and no difference was detected (AUC_0–16h_ differences 0.009, 95% CI −0.064 to 0.083, and 0.026, 95% CI −0.658 to 0.709; Holm-adjusted *p* = 1.00 in both cases). ArsMB expression therefore widens the window of peroxide stress that *E. coli* tolerates, rather than conferring total stress-proofing.

These findings show that arsMB expression enhances cell survival following lethal H_2_O_2_ exposure, consistent with ars1 and ars2 production conferring increased peroxide resistance. Indeed, the antioxidant activity of 5-alkylresorcinols (5-ARs) is attributed primarily to the two hydroxyl groups on the benzene ring, which play a significant role in radical scavenging and proton donation [104,105] (Fig 5A). Additionally, 5-ARs can integrate into the membrane and interact with phospholipids via hydrogen bonding, further enhancing their antioxidant effects [30,93,104] (Fig 5A). According to the “cutoff theory,” antioxidant efficacy increases with alkyl chain elongation until a hydrophobicity threshold is reached. Beyond this point, further extension of the alkyl chain reduces antioxidant capacity, likely because of diminished solubility or membrane compatibility [105].

Adipostatins and grevillol are alkylresorcinols that share a conserved resorcinol core and an alkyl chain at the C5 position. These compounds have been identified in both bacteria and plants [94,97,106]. Although reports on their biosynthesis via bacterial PKS III are limited, production has been documented in genera such as *Streptomyces* and *Azotobacter*. Bacterial adipostatins inhibit the enzymatic activity of asparaginyl-tRNA synthetase, leading to the death of the parasite *Brugia malayi* [99]. Although no studies have directly evaluated the antioxidant activity of Adipostatin A or grevillol, homologs of 5-ARs have been incorporated into lipid extracts from high-fiber cereal diets, suggesting a potential role in protecting against lipid oxidation [23,107,108]. The proposed antioxidant mechanism for phenolic lipids involves forming 1,2,4-trihydroxy-6-alkylbenzene as a primary oxidation product. This intermediate readily converts to *ortho*- and *para*-quinones, which possess enhanced radical-scavenging activity [109]. Beyond the phenolic moiety, the length of the aliphatic side chain significantly influences the antioxidant capacity of resorcinolic lipids [30]. For instance, orcinol requires concentrations approximately 10 times higher than those of 1,3-dihydroxy-5-pentadecylbenzene to achieve comparable antioxidant effects [105]. Similarly, olivetol (C_11_H_16_O_2_) exhibits considerable efficacy in mitigating the non-isothermal autoxidation of linolenic acid [110].

#### Impact on muralytic digestion with endolysins.

Bacteriophage-derived endolysins catalyze the cell wall peptidoglycan (PG) degradation (muralytic activity) to induce cell lysis [60,61]. The specific muralytic activity, defined as the decrease in ΔOD_600_ per minute per μg of endolysin (U/μg), is measured by the reduction in turbidity in endolysin-treated cells with exposed PG and an intact inner membrane [62]. The protective capacity of cell-associated phenolic lipids ars1 and ars2 on the *E. coli* host was assessed using three different endolysins: LysKZ144 [60], LysPA26 [61], and OsLys [62]. The muralytic activity of the three different endolysins against *E. coli* producing phenolic lipids ar1/ars2 (*E. coli/arsMB*) was evaluated by exogenous treatment (Fig 6B). The analysis revealed that in the non-producing control strain *E. coli* arsMB (-), all three endolysins catalyzed rapid turbidity decay; each turbidity trace was fitted individually to a one-phase exponential decay and summarised by its first-order lysis constant *k*, its residual turbidity plateau *P*, its initial rate, and its lysis AUC over 0–12 min (**S10 Table**). In the non-producing control strain, the three endolysins hydrolyzed the exposed peptidoglycan with k = 0.19–0.25 min − 1 (LysKZ144), 0.41–0.52 min − 1 (LysPA26), and 0.04–0.30 min − 1 (OsLys), reaching residual turbidity plateaux of 0.48–0.67, 0.56–0.75, and 0.23–0.63, respectively. In the ArsMB-producing strain, lysis was strongly attenuated at every enzyme concentration tested: the relative turbidity after 12 min remained ≥ 0.92 of its initial value, against 0.50–0.78 in the control, and the lysis AUC was reduced to ≤ 25% of the control value (LysKZ144 13–25%, LysPA26 ≤ 8%, OsLys 2–19%). Two-way ANOVA on the lysis AUC confirmed a dominant effect of the strain for the three enzymes (partial η^2^ = 0.998, 0.994 and 0.996 for LysKZ144, LysPA26 and OsLys; *p* < 0.0001) together with a significant strain × concentration interaction (partial η^2^ = 0.98, 0.87 and 0.97; *p* < 0.0001), *i.e.,* the difference between strains widens as more enzyme is added. All nine Holm-adjusted strain contrasts were significant, with large effect sizes (Hedges’ *g* = 9.0–88.8; **S10 Table**).

These results show that *E. coli* cells carrying ars1/ars2 are markedly, although not absolutely, protected from muralytic digestion by the three endolysins tested (Fig 5B). Previous molecular dynamics simulations support this observation, showing that ARs stabilize the membrane by significantly increasing lipid chain ordering [102,111]. This stabilization promotes a more compact, structured organization of phospholipids, thereby enhancing cellular resistance to external stressors, such as osmotic pressure fluctuations. Furthermore, interactions between the hydroxyl groups of resorcinol and the oxygen atoms of the phospholipid glycerol backbone induce membrane dehydration, reduce permeability, limit water entry, and promote lipid bilayer thickening [111].

On the other hand, the length of the alkyl side chain appears to have little influence on the positioning of these molecules within the bilayer, as in silico incorporation and reattachment simulations consistently show that ARs anchor in the same region of the bilayer [101,102]. From this point of view, if ars1/ars2 preserve cell turgor and increase membrane structural strength, they could help prevent endolysin-induced lysis. To date, no study has reported modifying the *E. coli* membrane using 5-ARs as a protective strategy against endolysins. However, the effects of ARs on the activity of hydrolytic enzymes, such as lysozyme and papain, have been documented [112,113]. Martirosova *et al.* (2015) demonstrated that methylresorcinol and 4-hexylresorcinol can modulate the functional activity of lysozyme and papain during chitosan hydrolysis. Petrovskii *et al.* (2009) found that alkylhydroxybenzenes (AHB) of alkyl chain of C7 enhance the activity of egg-white lysozyme against *Micrococcus luteus* and *Saccharomyces cerevisiae* cell wall; while AHB with a C12-alkyl chain can enhance or inhibit the same activity depending on its concentration [113]. ARs can interact with lytic enzymes, altering their conformation and substrate affinity and thereby enhancing resistance to denaturing conditions [114]. Additionally, ARs may bind directly to the substrate, inducing particular structural reorganization and modifying the number of accessible reactive sites [113,114]. Unlike the previously mentioned studies, in which ARs were applied exogenously, in the present study, we synthesized 5-ARs in the cytosol of the *E. coli/ars*MB system, *i.e*., within the cell. Therefore, it is possible that the 5-ARs were incorporated into the cytoplasmic membrane, altering its composition and protecting the cell from the lytic activity of endolysins. However, the extent and effectiveness of this protection remain to be determined experimentally. Furthermore, changes in membrane order and fluidity can also impact the activity of integral membrane proteins, suggesting that additional resistance mechanisms should be considered.

## Conclusions

This study reports the identification and functional characterization of ArsMB, a novel type III polyketide synthase (PKS) derived from the marine *Pontimonas* sp. Heterologous expression in *E. coli*, coupled with a precursor optimization strategy using glucose as the sole carbon source, demonstrated that *E. coli* carrying ArsMB produces cell-associated long-chain alkylresorcinols identified as 5-n-pentadecyl- and 5-n-tridecylresorcinol. Significantly, cellular accumulation of these amphipathic lipids increased host resistance to muralytic digestion by endolysins and to hydrogen peroxide. Collectively, these findings expand the known repertoire of PKSs from marine microbiomes.

## Supporting information

S1 Fig Algorithm for the search and identification of ArsMB from seawater microbial communities of the Baltic Sea. (PDF)

S2 Table Sequence identity of ArsMB with characterized bacterial type III PKSs. (PDF)

S3 Table List of enzymes used for the type III PKS phylogenetic analysis. (PDF)

S4 Table Complete GC-MS peak list of the Fast Blue B–positive fraction 14 from cell-associated products of E. coli/ArsMB. (PDF)

S5 Fig Superposition of the catalytic triad and the residues Arg280 and Arg317 in ArsMB and GCS. (PDF)

S6 Fig TLC analysis of ethyl acetate extracts from E. coli/pET-22b(+) and E. coli/ArsMB biomass. (PDF)

S7 Fig TLC analysis of ethyl acetate extracts from the culture medium and the biomass of E. coli/ArsMB. (PDF)

S8 Fig Individual replicate traces of the muralytic assays. (PDF)

S9 Table Strain contrasts on the derived parameters of the H_2_O_2_ and muralytic assays shown in Fig 6. (PDF)

S10 Table Per-well derived parameters, ANOVA tables, and linear mixed-effects model for the assays shown in Fig 6. (PDF)
