# Supplementary material for: A Baltic Sea microbiome-derived Type III polyketide synthase produces host-protective alkylresorcinols in E. coli
Source: PLoS One. 2026 Nov 3;21(11):e0361965. doi: 10.1371/journal.pone.0361965 (PMC13730376; doi:10.1371/journal.pone.0361965)

S1 Fig. Algorithm for search and identification of ArsMB from seawater microbial communities of the Baltic Sea.

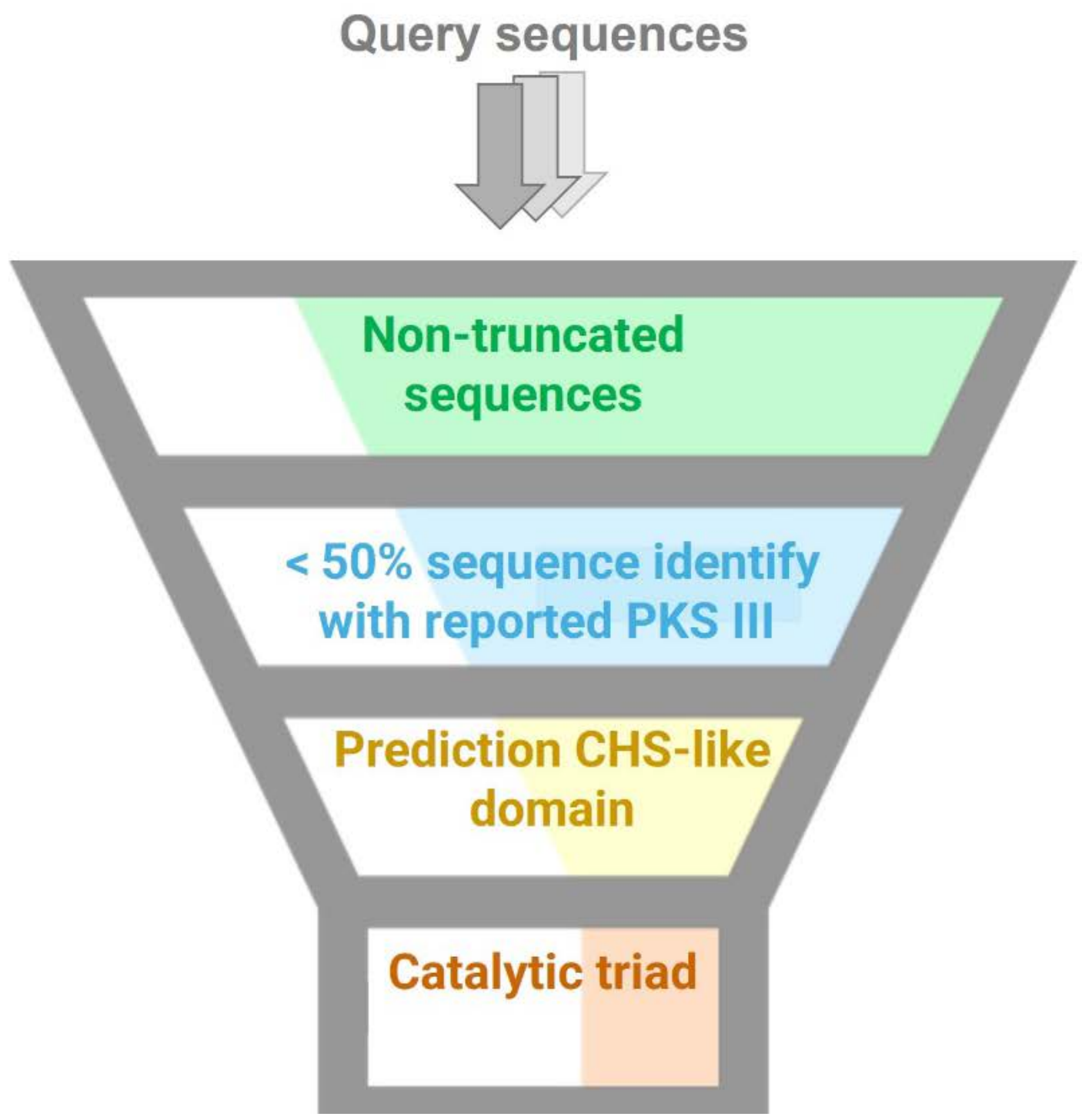

Supplement: Supplementary file 1 [file pone.0361965.s001.pdf]
