# Supplementary material for: A Baltic Sea microbiome-derived Type III polyketide synthase produces host-protective alkylresorcinols in E. coli
Source: PLoS One. 2026 Nov 3;21(11):e0361965. doi: 10.1371/journal.pone.0361965 (PMC13730376; doi:10.1371/journal.pone.0361965)
Supplement: Supplementary file 2 [file pone.0361965.s002.pdf]

S2 Table. Sequence identity with characterized bacterial type III PKS.

	Type III PKS	Strain/organism	NCBI ID	PDB	Identity (from 100 % coverage) (%)	Experimental characterization reference
	ArsMB	*Uncultured Pontimonas sp.*	KRO38894.1		100	This study
1	RppA	*Streptomyces griseus*	BAA33495.1		28.3	Funa et al., 1999
2	RppA	*Saccharopolyspora erythraea*	AAL78053.1		28.7	Cortes et al., 2002
3	RppB	*Streptomyces antibioticus*	BAB91444.1		31.9	Funa et al., 2002
4	RppA	*Streptomyces antibioticus*	BAB91443.1		30.1	
5	RppA	*Streptomyces lividans*	BAB91445.1		28.8	
6	THNS	*Streptomyces coelicolor A3(2)*	CAC01488.1	1U0M	28.8	Izumikawa et al., 2003
7	RppA	*Streptomyces peucetius subsp. caesius ATCC 27952*	ABY71276.1		42.6	Prasad et al., 2008
8	StTS	*Streptomyces toxytricini NRRL 15443*	AEO44526.1		32.2	Zeng et al., 2012
9	SoceCHS1	*Sorangium cellulosum So ce56*	AAZ93632.1		29.1	Gross et al., 2006
10	ThnA	*Nocardia sp. CS682*	QBS44862.1		27.2	Poudel et al., 2023
11	DpgA	*Amycolatopsis orientalis*	CAA11765.1		28.1	Chen et al., 2001
12	DpgA	*Amycolatopsis balhimycina DSM 5908*	CAC48378.1		28.1	Pfeifer et al., 2001
13	DpgA	*Streptomyces toyocaensis NRRL15009*	AAM80548.1		27.9	Pootoolal et al., 2002

14	Ken2	*Streptomyces violaceoruber 3844-33C*	CAQ52620.1		28.6	Wenzel et al., 2008
15	SCO7671	*Streptomyces coelicolor*	WP_011031818.1		38.6	Grüschow et al., 2007
16	DpyA	*Streptomyces reveromyceticus*	BAQ19510.1		30.3	Aizawa et al., 2014
16	Gcs	*Streptomyces coelicolor A3(2)*	CAB94644.1	3V7I	24.3	Song et al., 2006
17	ArsC	*Azotobacter vinelandii*	ZP_00418326.1	3VS8	21.0	Funa et al., 2006
18	ArsB	*Azotobacter vinelandii*	AGK17581.1		21.0	Funa et al., 2006
19	PKS18	*Mycobacterium tuberculosis H37Rv*	P9WPF1.1	1TED	28.8	Saxena et al., 2003
20	PKS11	*Mycobacterium tuberculosis H37Rv*	P9WPF3.1	4JAO	46.3	Saxena et al., 2003
21	PKS10	*Mycobacterium marinum M*	ACC40920.1		43.5	Parvez et al., 2018
22	PKS11	*Mycobacterium marinum M*	ACC40924.1		45.5	Parvez et al., 2018
23	PKS10	*Mycolicibacterium smegmatis*	ABK69605.1		44.0	Anand et al., 2015
24	PhlD	*Pseudomonas protegens Pf-5*	AAY95147.1		30.7	Anhkar et al., 2005
25	PhlD	*Pseudomonas fluorescens Q2-87*	AAB48106.1		30.2	Bangera and Thomashow, 1999
26	BpsA	*Bacillus subtilis subsp. subtilis str. 168*	WP_327840277.1		30.6	Nakano et al., 2009
27	RpsA	*Rhodospirillum centenum*	ACI98675.1		33.7	Awakawa et al., 2013
28	AgqA	*Actinoplanes missouriensis*	BAL92976.1		31.5	Awakawa et al., 2011
29	FtpA	*Myxococcus xanthus*	ABF87000.1		44.5	Hayashi et al., 2011

30	SrsA	*Streptomcyes griseus NBRC 13350*	BAG17301.1		48.0	Funabashi et al., 2008
31	CylI	*Cylindrospermum licheniforme UTEX B 2014*	AFV96143.1		31.3	Nakamura et al., 2012
32	HidC	*Cyanobium sp. LEGE06113*	QBC65480.1		30.2	Costa et al., 2018
33	LpmD	*Streptomyces sp. SN-1061M*	ADC96652.1		27.8	Kaysser et al., 2010
34	Cpz6	*Streptomyces sp. MK730–62F2*	NEE14180.1*		28.3	Tang et al., 2013
35	VioA	*Streptomyces somaliensis SCSIO ZH66*	AMN09000.1		27.3	Hou et al., 2018
36	CwoePKS	*Conexibacter woesei DSM 14684*	ADB53388.1		28.2	Wiker et al., 2019
37	LosA	*Micromonospora endolithica*	QZS07521.1		31.1	Lash et al., 2020
38	CepA	*Candidatus Entotheonella sp.*	QEO32865.1		30.9	Reiter et al., 2020
39	Kmy18	*Verrucosispora sp. SCSIO 07399*	UHY14145.1		29.6	Chen et al., 2021
40	Adp	*Streptomyces davawensis DSM101723*	CCK30296.1		30.1	Hou et al., 2021
41	DquA	*Dendrosporobacter quercicolus DSM1736*	WP_092073450.1		35.5	Bredy et al., 2022
