# Supplementary material for: A Baltic Sea microbiome-derived Type III polyketide synthase produces host-protective alkylresorcinols in E. coli
Source: PLoS One. 2026 Nov 3;21(11):e0361965. doi: 10.1371/journal.pone.0361965 (PMC13730376; doi:10.1371/journal.pone.0361965)
Supplement: Supplementary file 3 [file pone.0361965.s003.pdf]

S3 Table. List of enzymes used for type III PKS phylogenetic analysis.

	Type III PKS	Strain/organism	Taxonomic affiliation	NCBI ID	Product/pathway	Intramolecular cyclization type	Experimental characterization reference
**Bacterial type III PKS**							
	**ArsMB**	*Pontimonas sp.*	Actinobacteria	KRO38894.1	Alkylresorcinols	Aldol	This study
1	RppA	*Streptomyces griseus*	Actinobacteria	BAA33495.1	1,3,6,8-tetrahydroxynaphthalene (THN)/ melanins and naphthoquinone ring derivates	Dual intramolecular Claisen and aldol condensations	Funa et al., 1999
2	RppA	*Saccharopolyspora erythraea*	Actinobacteria	AAL78053.1	THN/ melanins and naphthoquinone ring derivates	Dual intramolecular Claisen and aldol condensations	Cortes et al., 2002
3	RppB	*Streptomyces antibioticus*	Actinobacteria	BAB91444.1	THN/ melanins and naphthoquinone ring derivates	Dual intramolecular Claisen and aldol condensations	Funa et al., 2002
4	RppA	*Streptomyces antibioticus*	Actinobacteria	BAB91443.1	THN/ melanins and naphthoquinone ring derivates	Dual intramolecular Claisen and aldol condensations	
5	RppA	*Streptomyces lividans*	Actinobacteria	BAB91445.1	THN/ melanins and naphthoquinone ring derivates	Dual intramolecular Claisen and aldol condensations	
6	THNS	*Streptomyces coelicolor A3(2)*	Actinobacteria	CAC01488.1	THN/ melanins and naphthoquinone ring derivates	Dual intramolecular Claisen and aldol condensations	Izumikawa et al., 2003

7	StTS	*Streptomyces toxytricini NRRL 15443*	Actinobacteria	AEO44526.1	THN/ melanins and naphthoquinone ring derivates	Dual intramolecular Claisen and aldol condensations	Zeng et al., 2012
8	SoceC HS1	*Sorangium cellulosum So ce56*	Myxococcia	AAZ93632.1	THN/ melanins and naphthoquinone ring derivates	Dual intramolecular Claisen and aldol condensations	Gross et al., 2006
9	ThnA	*Nocardia sp. CS682*	Actinobacteria	QBS44862.1	THN/IBR-3	Dual intramolecular Claisen and aldol condensations	Poudel et al., 2023
10	DpgA	*Amycolatopsis orientalis*	Actinobacteria	CAA11765.1	3.5-Dihydroxyphenylacetyl-CoA (DPA-CoA)/ vancomycin,	Aldol	Chen et al., 2001
11	DpgA	*Amycolatopsis balhimycina DSM 5908*	Actinobacteria	CAC48378.1	DPA-CoA/ balhimycin	Aldol	Pfeifer et al., 2001
12	DpgA	*Streptomyces toyocaensis NRRL15009*	Actinobacteria	AAM80548.1	DPA-CoA/ A47934	Aldol	Pootoolal et al., 2002
13	Ken2	*Streptomyces violaceoruber 3844-33C*	Actinobacteria	CAQ52620.1	DPA-CoA/ kendomycin	Aldol	Wenzel et al., 2008
14	SCO76 71	*Streptomyces coelicolor*	Actinobacteria	WP_011031818 .1	2-pyrones	Lactonization	Grüschow et al., 2007
15	Gcs	*Streptomyces coelicolor A3(2)*	Actinobacteria	CAB94644.1	Germicidin A-D (2-pyrones)	Lactonization	Song et al., 2006
16	ArsC	*Azotobacter vinelandii*	γ-proteobacteria	ZP_00418326.1	Tri and tetra alkylpyrones	Lactonization	Funa et al., 2006
17	ArsB	*Azotobacter vinelandii*	γ-proteobacteria	AGK17581.1	Alkyresorcinols	Aldol	Funa et al., 2006
18	PKS18	*Mycobacterium tuberculosis H37Rv*	Actinobacteria	P9WPF1.1	Tri and tetra-alkylpyrones (C12-C20)	Lactonization	Saxena et al., 2003
19	PKS11	*Mycobacterium tuberculosis H37Rv*	Actinobacteria	P9WPF3.1	Methyl-branched alkylpyrones	Lactonization	Saxena et al., 2003
20	PKS10	*Mycobacterium marinum M*	Actinobacteria	ACC40920.1	Methylated alkylpyrones	Lactonization	Parvez et al., 20185
21	PKS11	*Mycobacterium marinum M*	Actinobacteria	ACC40924.1	Methylated alkylpyrones,methylated	Lactonization, Aldol, Claisen	Parvez et al., 2018

					alkyresorcinols and methylated acyl-phloroglucinols		
22	PKS10	*Mycolicibacterium smegmatis*	Actinobacteria	ABK69605.1	Alkylresorcinols/ polyketide quinones (PkQs)	Aldol	Anand et al., 2015
23	PhlD	*Pseudomonas protegens Pf-5*	γ-proteobacteria	AAY95147.1	Phloroglucinol/ Diacetylphloroglucinol (2,4-DAPG)	Claisen	Anchkar et al., 2005
24	PhlD	*Pseudomonas fluorescens Q2-87*	γ-proteobacteria	AAB48106.1	Phloroglucinol/2.4-DAPG	Claisen	Bangera and Thomashow, 1999
25	BpsA	*Bacillus subtilis subsp. subtilis str. 168*	Bacilli	AAA96613.1	Tri and tetra-pyronces, alkylresorcinols	Lactonization, aldol	Nakano et a., 2009
26	RpsA	*Rhodospirillum centenum*	α-proteobacteria	ACI98675.1	Methylated tetraketide pyrones, resorcinols and Resorcylic acids	Lactonization, aldol	Awakawa et al., 2013
27	AgqA	*Actinoplanes missouriensis*	Actinobacteria	BAL92976.1	Alkylresorcinols, alkylresorcilyc acid	Aldol	Awakawa et al., 2011
28	FtpA	*Myxococcus xanthus*	Myxococcia	ABF87000.1	Alkylresorcylic acids	Aldol	Hayashi et al., 2011
29	SrsA	*Streptomcyes griseus NBRC 13350*	Actinomycetota	BAG17301.1	Alkylresorcylic acids	Aldol	Funabashi et al., 2008
30	CylI	*Cylindrospermum licheniforme UTEX B 2014*	Cyanobacteria	AFV96143.1	Alkylresorcinols/ Cylindrocyclophanes.	Aldol	Nakamura et a., 2012
31	HidC	*Cyanobium sp. LEGE06113*	Cyanobacteria	QBC65480.1	Alkylresorcinols/ Hierrdins B and C	Aldol	Costa et al., 2018
32	LpmD	*Streptomyces sp. SN-1061M*	Actinobacteria	ADC96652.1	Presulficidins/Liposidomycin	Lactonization	Kaysser et al., 2010
33	Cpz6	*Streptomyces sp. MK730–62F2*	Actinobacteria	NEE14180.1*	Presulficidins/Caprazamycins	Lactonization	Tang et al., 2013
34	VioA	*Streptomyces somaliensis SCSIO ZH66*	Actinobacteria	AMN09000.1	Violapyrones	Lactonization	Hou et a., 2018
35	LosA	*Micromonospora endolithica*	Actinobacteria	QZS07521.1	Loseolamycin	Aldol	Lash et al., 2020
36	CepA	*Candidatus Entotheonella sp.*	Nitrospiria	QEO32865.1	Alkyresorcinols, alkylpyrones	Aldol, lactonization	Reiter et al., 2020

37	Kmy18	*Verrucosispora sp. SCSIO 07399*	Actinobacteria	UHY14145.1	DPA-CoA/ kendomycin	Aldol	Chen et al., 2021
38	Adp	*Streptomyces davawensis DSM101723*	Actinobacteria	CCK30296.1	Adipostatins	Aldol	Hou et al., 2021
39	DquA	*Dendrosporobacter quercicolus DSM1736*	Negativicutes	WP_092073450 .1	Tri and tetra-akylpyrones	Lactonization	Bredy et al., 2022
40	PKS18-like	*Mycobacterium bourgelatii*	Actinobacteria	GFG90093.1	Alpha-pyrone synthesis polyketide synthase-like Pks18 (inferred from homology)	Lactonization (inferred from homology)	Matsumoto et al., 2019
41	PKS III	*Methylocaldum sp*	γ-proteobacteria	WP_347257714 .1	Putative PKS III	-	-
42	PKS III	*Algoriphagus iocasae*	Cytophagia	WP_184494880 .1	Putative naringenin-chalcone synthase/ polyketide biosynthetic process	-	Funa et al., 1999

Fungal type III PKS

43	**PKS III Nc (ORAS)**	*Neurospora crassa OR74A*	Ascomycota	XP_960427.1	2′-oxoalkylresorcylic acid, tri and tetra-pyrones, tetra and penta-alkylresorcinols	Aldol, Lactonization	Funa et al., 2007 Goyal et al., 2008
44	**AnPKS**	*Aspergillus niger CBS 513.88*	Ascomycota	XP_001390871. 1	Tri and tetraketide pyrones, alkylresorcinols, Protocatechuic acid	Aldol, lactonization	Li et al., 2011
45	**BPKS**	*Botrytis cinerea B05.10*	Ascomycota	XP_001555327. 1	Tri and tetraketide pyrones, pentaketido-resorcinols,	Lactonization, aldol	Jeya et a., 2012
46	**CsyA**	*Aspergillus oryzae RIB40*	Ascomycota	BAD97390.1	Tri and tetraketide pyrones	Lactonization	Yu et al., 2010
47	**CsyB**	*Aspergillus oryzae RIB40*	Ascomycota	Q2U852.1	Acetolated alkylpyrones	Lactonization	Hashimoto et al., 2013
48	**PKS2**	*Sporotrichum laxum ATCC 15155*	Basidiomycota	AMW87980.1	Alkyresorcinols, alkylresorcinolic acid/ Spirolaxine	Aldol	Sun et al., 2016
49	**SsArs**	*Shiraia sp. slf14*	Ascomycota	AXB87495.1	Alk(en)ylresorcinols	Aldol	Yan et al., 2018

50	**PspB**	*Penicillium soppi*	Ascomycota	P0DUK2.1	1,3,5-trisubstituted alkylresorcinol	Aldol	Kaneko et al., 2019
51	**CtPKS**	*Chaetomium thermophilum*	Ascomycota	XP_006694643.1	Tri and tetraketide pyrones, resorcinols, resorcylic acids	Lactonization, aldol	Ramakrishnan et al., 2018
52	**SmPKS**	*Sordaria macrospora*	Ascomycota	XP_003353120.1	Tri and tetraketide pyrones, resorcinols, resorcylic acids	Lactonization, aldol	Ramakrishnan et al., 2018
53	**FiPKS**	*Fusarium incarnatum*	Ascomycota	AUO79642.1	Pyrones, resorcinols	Lactonization, aldol	Manoharan et al., 2019
54	**Std**	*Naganishia uzbekistanensis strain Mo29*	Basidiomycota	QRF93456.1	Tri and tetraketide pyrones, alkylresorcinols	Lactonization, aldol	Martinelli et al., 2020

Plant type III PKS

55	**CHS**	*Medicago sativa*	Streptophyta	P30074.1	Naringenin chalcone	Claisen	Ferrer et al., 1999
56	**VPS**	*Humulus lupulus*	Streptophyta	O80400.1	Phlorisovalerophenone	Claisen	Paniego et al., 1999<br>Okada and Ito, 2001
57	**ACS**	*Huperzia serrata*	Streptophyta	ABI94386.1	1,3-Dihydroxy-Nmethylacridone, 4-methoxy-2′,4′,6′-trihydroxychalcone	Claisen	Wanibuchi et al., 2007
58	**STS**	*Pinus sylvestris*	Streptophyta	Q02323.1	Pinosylvin	Aldol	Schanz et al., 1992
59	**STS**	*Arachis hypogaea*	Streptophyta	BAA78617.1	Resveratrol	Aldol	Shomura et al., 2005
60	**BBS**	*Phalaenopsis sp.*	Streptophyta	P53416.1	3,3’,5-Trihydroxybibenzyl	Aldol	Preisig-Müller et al., 1995
61	**ORS**	*Rhododendron dauricum*	Streptophyta	BAV83003.1	Orcinol, orsellinic acid	Aldol	Taura et al., 2009
62	**OLS**	*Cannabis sativa*	Streptophyta	BAG14339.1	Olivetol	Aldol	Taura et al., 2009
63	**2PS**	*Gerbera hybrid*	Streptophyta	CAA86219.2	2-pyrones, triacetolactones	Lactonization,	Eckermann et al., 1998
64	**PstrCHS2**	*Pinus strobus*	Streptophyta	CAA05214.1	Methyl-pyrone	Lactonization,	Schöder et al., 1998
65	**PKS-A**	*Arabidopsis thaliana*	Streptophyta	O23674.1	Tri and tetraketide-Alkylpyrones	Lactonization,	Mizuuchi et al., 2008

66	**PKS-B**	*Arabidopsis thaliana*	Streptophyta	Q8LDM2.1	Tri and tetraketide-Alkylpyrones	Lactonization,	Mizuuchi et al., 2008
67	**DCS**	*Curcuma longa*	Streptophyta	BAH56225.1	Diketides-CoA, feruloyldiketide-CoA, coumarolydiketide-CoA	No cyclization	Katsuyama et al., 2009
68	**CURS**	*Curcuma longa*	Streptophyta	BAH56226.1	Curcuminoids	No cyclization	Katsuyama et al., 2009
69	**QNS**	*Aegle marmelos*	Streptophyta	AGE44110.1	Quinolones, acridones, benzalacetones	Nitrogen-carbon-forming, no cyclization	Resmi et al., 2013
70	**ALS**	*Rheum palmatum*	Streptophyta	AAS87170.1	Aloesone	C8-C13 aldol cyclization with an additional second ring formation	Abe et al., 2004
71	**PCS**	*Aloe arborescens*	Streptophyta	AAX35541.1	5,7-Dihydroxy-2-methylchromone	Claisen and probable spontaneous formation of a second ring	Abe et al., 2005
72	**ScARS**	*Secale cereale*	Streptophyta	QHR78940.1	Alkylresorcinols, 2-oxo-alkylresorcinols	Aldol	Targońska-Karasek et al., 2023

Protist Type III PKS

73	**Esi-PKS1**	*Ectocarpus siliculosus*	Stramenopiles	CBN76919.1	(Acyl)phloroglucinols, tetraketide pyrones	Claisen, lactonization	Meslet-Cladière et al., 2013
74	**SbPKS**	*Sargassum binderi*	Stramenopiles	ADK13089.1	Tri and tetraketide pyrones	Lactonization,	Baharum et al., 2011
75	**SfuPKS1**	*Sargassum fusiforme*	Stramenopiles	UMB20798.1	Tri and tetraketide pyrones, phloroglucinol derivatives	Lactonization, Claisen	Zhao et al., 2021
76	**StlB**	*Dictyostelium discoideum*	Amoebozoa	Q54FI3.1	Acylphloroglucinols/DIF-1	Claisen	Austin et al., 2006

Other type III PKS

77	S9PKS	*uncultured organism*		QSV39300.1	Tri and tetra-alkylpyrones	Lactonization	Thenmozhi Kulasekaran et al., 2024

Bacterial KAS Ⅲ (outgroup)

78	**FabH**	*Escherichia coli*	γ-proteobacteria	AAA23749.1	Fatty acid biosynthesis		Heath and Rock, 1996
79	**FabH**	*Mycobacterium tuberculosis H37Rv*	Actinobacteria	CCP43271.1	Fatty acid biosynthesis		Choi et al., 2000
80	**FabH**	*Streptomyces glaucescens*	Actinobacteria	AAA99447.1	Fatty acid biosynthesis		Han et al., 1998
81	**FabH**	*Bacillus subtilis 168*	Bacilli	CAB12974.1	Fatty acid biosynthesis		Choi et al., 2000b
82	**FabH**	*Salmonella enterica*	γ-proteobacteria	AAL20122.1	Fatty acid biosynthesis		McClelland et al., 2001
83	**FabH**	*Geobacillus kaustophilus HTA426*	Bacilli	BAD75089.1	Fatty acid biosynthesis		Takami et al., 2004
84	**FabH**	*Xanthomonas campestris*	γ-proteobacteria	AAY50273.1	Fatty acid biosynthesis		Qian et al., 2005
85	**FabH**	Streptomyces lividans	Actinobacteria	AAG30195.1	Fatty acid biosynthesis		Marti et al., 2000
86	**FabH**	*Streptomyces peucetius*	Actinobacteria	AAA65208.1	Daunorubicin biosynthesis		Grimm et al., 1994

REFERENCES

N. Funa, Y. Ohnishi, I. Fujli, M. Shibuya, Y. Ebizuka, and S. Horinouchi, "A new pathway for polyketide synthesis in microorganisms," *Nat. 1999 4006747*, vol. 400, no. 6747, pp. 897–899, Aug. 1999, doi: 10.1038/23748.

J. Cortés, J. Velasco, G. Foster, A. P. Blackaby, B. A. M. Rudd, and B. Wilkinson, "Identification and cloning of a type III polyketide synthase required for diffusible pigment biosynthesis in *Saccharopolyspora erythraea*," *Mol. Microbiol.*, vol. 44, no. 5, pp. 1213–1224, 2002, doi: 10.1046/J.1365-2958.2002.02975

N. Funa, Y. Ohnishi, Y. Ebizuka, and S. Horinouchi, "Alteration of reaction and substrate specificity of a bacterial type III polyketide synthase by site-directed mutagenesis," *Biochem. J.*, vol. 367, no. Pt 3, pp. 781–789, Nov. 2002, doi: 10.1042/BJ20020953.

M. Izumikawa *et al.*, "Expression and characterization of the type III polyketide synthase 1,3,6,8-tetrahydroxynaphthalene synthase from *Streptomyces coelicolor* A3(2)," *J. Ind. Microbiol. Biotechnol.*, vol. 30, no. 8, pp. 510–515, Aug. 2003, doi: 10.1007/S10295-003-0075-8.

G. Prasad Ghimire, J. Kyung Sohng, T.-J. Oh, and K. Liou, "Identification of a cryptic type III polyketide synthase (1, 3, 6, 8-tetrahydroxynaphthalene synthase) from *Streptomyces peucetius* ATCC 27952.," *Res. Ghimire, TJ Oh, K Liou, JK SohngMolecules Cells (Springer Sci. Bus. Media BV), 2008,* pp. 362–367, 2008, doi: 10.1016/S1016-8478(23)14009-X.

J. Zeng, R. Decker, and J. Zhan, "Biochemical characterization of a type III polyketide biosynthetic gene cluster from *Streptomyces toxytricini*," *Appl. Biochem. Biotechnol.*, vol. 166, no. 4, pp. 1020–1033, Feb. 2012, doi: 10.1007/S12010-011-9490-X/METRICS.

F. Gross *et al.*, "Bacterial type III polyketide synthases: phylogenetic analysis and potential for the production of novel secondary metabolites by heterologous expression in

pseudomonads," *Arch. Microbiol.*, vol. 185, no. 1, pp. 28–38, Mar. 2006, doi: 10.1007/S00203-005-0059-3.
P. B. Poudel, R. T. Magar, A. F. Bridget, and J. K. Sohng, "Identification of 1,3,6,8-Tetrahydroxynaphthalene Synthase (ThnA) from *Nocardia* sp. CS682," *J. Microbiol. Biotechnol.*, vol. 33, no. 7, p. 949, Jul. 2023, doi: 10.4014/JMB.2303.03008.
H. Chen, C. C. Tseng, B. K. Hubbard, and C. T. Walsh, "Glycopeptide antibiotic biosynthesis: Enzymatic assembly of the dedicated amino acid monomer (S)-3,5-dihydroxyphenylglycine," *Proc. Natl. Acad. Sci. U. S. A.*, vol. 98, no. 26, pp. 14901–14906, Dec. 2001, doi: 10.1073/PNAS.221582098/ASSET/AA410625-4349-47AD-92F5-BDE612DB00C2/ASSETS/GRAPHIC/PQ0125820005.JPEG.
V. Pfeifer *et al.*, "A Polyketide Synthase in Glycopeptide Biosynthesis: the biosynthesis of the non-proteinogenic amino acid (s)-3,5-dihydroxyphenylglycine," *J. Biol. Chem.*, vol. 276, no. 42, pp. 38370–38377, Oct. 2001, doi: 10.1074/JBC.M106580200.
J. Pootoolal *et al.*, "Assembling the glycopeptide antibiotic scaffold: The biosynthesis of from *Streptomyces toyocaensis* NRRL15009," *Proc. Natl. Acad. Sci. U. S. A.*, vol. 99, no. 13, p. 8962, Jun. 2002, doi: 10.1073/PNAS.102285099.
S. C. Wenzel, H. B. Bode, I. Kochems, and R. Müller, "A type I/type III polyketide synthase hybrid biosynthetic pathway for the structurally unique ansa compound kendomycin," *Chembiochem*, vol. 9, no. 16, pp. 2711–2721, 2008, doi: 10.1002/CBIC.200800456.
S. Grüschow, T. J. Buchholz, W. Seufert, J. S. Dordick, and D. H. Sherman, "Substrate profile analysis and ACP-mediated acyl transfer in *Streptomyces coelicolor* Type III polyketide synthases," *Chembiochem*, vol. 8, no. 8, pp. 863–868, May 2007, doi: 10.1002/CBIC.200700026.
T. Aizawa *et al.*, "Alkyldihydropyrones, new polyketides synthesized by a type III polyketide synthase from *Streptomyces reveromyceticus*," *J. Antibiot. 2014 6712*, vol. 67, no. 12, pp. 819–823, Jul. 2014, doi: 10.1038/ja.2014.80.
L. Song *et al.*, "Type III polyketide synthase β-ketoacyl-ACP starter unit and ethylmalonyl-CoA extender unit selectivity discovered by *Streptomyces coelicolor* genome mining," *J. Am. Chem. Soc.*, vol. 128, no. 46, pp. 14754–14755, Nov. 2006, doi: 10.1021/JA065247W
N. Funa, H. Ozawa, A. Hirata, and S. Horinouchi, "Phenolic lipid synthesis by type III polyketide synthases is essential for cyst formation in *Azotobacter vinelandii*," *Proc. Natl. Acad. Sci.*, vol. 103, no. 16, pp. 6356–6361, Apr. 2006, doi: 10.1073/PNAS.0511227103.
P. Saxena, G. Yadav, D. Mohanty, and R. S. Gokhale, "A New Family of Type III Polyketide Synthases in *Mycobacterium tuberculosis*," *J. Biol. Chem.*, vol. 278, no. 45, pp. 44780–44790, Nov. 2003, doi: 10.1074/JBC.M306714200.
A. Parvez, S. Giri, G. R. Giri, M. Kumari, R. Bisht, and P. Saxena, "Novel Type III Polyketide Synthases Biosynthesize Methylated Polyketides in *Mycobacterium marinum*," *Sci. Rep.*, vol. 8, no. 1, Dec. 2018, doi: 10.1038/s41598-018-24980-1.
A. Anand *et al.*, "Polyketide Quinones Are Alternate Intermediate Electron Carriers during Mycobacterial Respiration in Oxygen-Deficient Niches," *Mol. Cell*, vol. 60, no. 4, pp. 637–650, Nov. 2015, doi: 10.1016/J.MOLCEL.2015.10.016.
J. Achkar, M. Xian, H. Zhao, and J. W. Frost, "Biosynthesis of phloroglucinol," *J. Am. Chem. Soc.*, vol. 127, no. 15, pp. 5332–5333, Apr. 2005, doi: 10.1021/JA042340G.
M. G. Bangera and L. S. Thomashow, "Identification and characterization of a gene cluster for synthesis of the polyketide antibiotic 2,4-diacetylphloroglucinol from *Pseudomonas fluorescens* Q2-87," *J. Bacteriol.*, vol. 181, no. 10, pp. 3155–3163, 1999, doi: 10.1128/JB.181.10.3155-3163.1999.
C. Nakano, H. Ozawa, G. Akanuma, N. Funa, and S. Horinouchi, "Biosynthesis of aliphatic polyketides by type III polyketide synthase and methyltransferase in *Bacillus subtilis*," *J. Bacteriol.*, vol. 191, no. 15, pp. 4916–4923, Aug. 2009, doi: 10.1128/JB.00407-09.
T. Awakawa *et al.*, "4-Hydroxy-3-methyl-6-(1-methyl-2-oxoalkyl)pyran-2-one Synthesis by a Type III Polyketide Synthase from *Rhodospirillum centenum*," *ChemBioChem*, vol. 14, no. 8, pp. 1006–1013, May 2013, doi: 10.1002/CBIC.201300066.
T. Awakawa, N. Fujita, M. Hayakawa, Y. Ohnishi, and S. Horinouchi, "Characterization of the biosynthesis gene cluster for alkyl-O-dihydrogeranyl-methoxyhydroquinones in *Actinoplanes missouriensis*," *Chembiochem*, vol. 12, no. 3, pp. 439–448, Feb. 2011, doi: 10.1002/CBIC.201000628.
T. Hayashi, Y. Kitamura, N. Funa, Y. Ohnishi, and S. Horinouchi, "Fatty acyl-AMP ligase involvement in the production of alkylresorcylic acid by *a Myxococcus xanthus* type III polyketide synthase," *Chembiochem*, vol. 12, no. 14, pp. 2166–2176, Sep. 2011, doi: 10.1002/CBIC.201100344.
M. Funabashi, N. Funa, and S. Horinouchi, "Phenolic lipids synthesized by type III polyketide synthase confer penicillin resistance on *Streptomyces griseus*," *J. Biol. Chem.*, vol. 283, no. 20, pp. 13983–13991, May 2008, doi: 10.1074/JBC.M710461200.
H. Nakamura, H. A. Hamer, G. Sirasani, and E. P. Balskus, "Cylindrocyclophane biosynthesis involves functionalization of an unactivated carbon center," *J. Am. Chem. Soc.*, vol. 134, no. 45, pp. 18518–18521, Nov. 2012, doi: 10.1021/JA308318P.
M. Costa *et al.*, "Structure, synthesis of hierridin C and discovery of prevalent alkylresorcinol biosynthesis in picocyanobacteria," 2018.

L. Kaysser, S. Siebenberg, B. Kammerer, and B. Gust, "Analysis of the liposidomycin gene cluster leads to the identification of new caprazamycin derivatives," *Chembiochem*, vol. 11, no. 2, pp. 191–196, Jan. 2010, doi: 10.1002/CBIC.200900637.

X. Tang, K. Eitel, L. Kaysser, A. Kulik, S. Grond, and B. Gust, "A two-step sulfation in antibiotic biosynthesis requires a type III polyketide synthase," *Nat. Chem. Biol. 2013 910*, vol. 9, no. 10, pp. 610–615, Aug. 2013, doi: 10.1038/nchembio.1310.

L. Hou, H. Huang, H. Li, S. Wang, J. Ju, and W. Li, "Overexpression of a type III PKS gene affording novel violapyrones with enhanced anti-influenza A virus activity," *Microb. Cell Fact.*, vol. 17, no. 1, pp. 1–11, Apr. 2018, doi: 10.1186/S12934-018-0908-9.

F. Wiker *et al.*, "Identification of Novel α-Pyrones from *Conexibacter woesei* Serving as Sulfate Shuttles," *ACS Chem. Biol.*, vol. 14, no. 9, pp. 1972–1980, Sep. 2019, doi: 10.1021/ACSCHEMBIO.9B00455.

C. Lasch, N. Gummerlich, M. Myronovskyi, A. Palusczak, J. Zapp, and A. Luzhetskyy, "Loseolamycins: A Group of New Bioactive Alkylresorcinols Produced after Heterologous Expression of a Type III PKS from *Micromonospora endolithica*," *Mol. 2020, Vol. 25, Page 4594*, vol. 25, no. 20, p. 4594, Oct. 2020, doi: 10.3390/MOLECULES25204594.

S. Reiter, J. K. B. Cahn, V. Wiebach, R. Ueoka, and J. Piel, "Characterization of an Orphan Type III Polyketide Synthase Conserved in Uncultivated 'Entotheonella' Sponge Symbionts," *Chembiochem*, vol. 21, no. 4, pp. 564–571, Feb. 2020, doi: 10.1002/CBIC.201900352.

J. Chen, S. Zhang, Y. Chen, X. Tian, Y. Gu, and J. Ju, "Identification and Heterologous Expression of the Kendomycin B Biosynthetic Gene Cluster from *Verrucosispora* sp. SCSIO 07399," *Mar. Drugs*, vol. 19, no. 12, Dec. 2021, doi: 10.3390/MD19120673.

L. Hou *et al.*, "Discovery of anti-infective adipostatins through bioactivity-guided isolation and heterologous expression of a type III polyketide synthase," *Bioorg. Chem.*, vol. 112, Jul. 2021, doi: 10.1016/J.BIOORG.2021.104925.

F. Bredy, K. Ishida, and C. Hertweck, "A Type III Polyketide Synthase Specific for Sporulating Negativicutes is Responsible for Alkylpyrone Biosynthesis," *Chembiochem*, vol. 23, no. 21, Nov. 2022, doi: 10.1002/CBIC.202200431.

Y. Matsumoto *et al.*, "Comprehensive subspecies identification of 175 nontuberculous mycobacteria species based on 7547 genomic profiles," *Emerg. Microbes Infect.*, vol. 8, no. 1, p. 1043, Jan. 2019, doi: 10.1080/22221751.2019.1637702.

N. Funa, T. Awakawa, and S. Horinouchi, "Pentaketide resorcylic acid synthesis by type III polyketide synthase from *Neurospora crassa*," *J. Biol. Chem.*, vol. 282, no. 19, pp. 14476–14481, May 2007, doi: 10.1074/JBC.M701239200.

A. Goyal *et al.*, "Structural insights into biosynthesis of resorcinolic lipids by a type III polyketide synthase in *Neurospora crassa*," *J. Struct. Biol.*, vol. 162, no. 3, pp. 411–421, Jun. 2008, doi: 10.1016/J.JSB.2008.02.009.

J. Li, Y. Luo, J. K. Lee, and H. Zhao, "Cloning and characterization of a type III polyketide synthase from *Aspergillus niger*," *Bioorg. Med. Chem. Lett.*, vol. 21, no. 20, pp. 6085–6089, Oct. 2011, doi: 10.1016/J.BMCL.2011.08.058.

M. Jeya, T. S. Kim, M. K. Tiwari, J. Li, H. Zhao, and J. K. Lee, "The Botrytis cinerea type III polyketide synthase shows unprecedented high catalytic efficiency toward long chain acyl-CoAs," *Mol. Biosyst.*, vol. 8, no. 11, pp. 2864–2867, Oct. 2012, doi: 10.1039/C2MB25282A.

D. Yu, J. Zeng, D. Chen, and J. Zhan, "Characterization and reconstitution of a new fungal type III polyketide synthase from *Aspergillus oryzae*," *Enzyme Microb. Technol.*, vol. 46, no. 7, pp. 575–580, Jun. 2010, doi: 10.1016/J.ENZMICTEC.2010.02.011.

M. Hashimoto, S. Ishida, Y. Seshime, K. Kitamoto, and I. Fujii, "Aspergillus oryzae type III polyketide synthase CsyB uses a fatty acyl starter for the biosynthesis of csypyrone B compounds.," *Bioorg. Med. Chem. Lett.*, vol. 23, no. 20, pp. 5637–5640, Aug. 2013, doi: 10.1016/J.BMCL.2013.08.036.

L. Sun *et al.*, "Identification of a type III polyketide synthase involved in the biosynthesis of spirolaxine," *Appl. Microbiol. Biotechnol.*, vol. 100, no. 16, pp. 7103–7113, Aug. 2016, doi: 10.1007/S00253-016-7444-5.

H. Yan *et al.*, "Identification and heterologous reconstitution of a 5-alk(en)ylresorcinol synthase from endophytic fungus *Shiraia* sp. Slf14," *J. Microbiol.*, vol. 56, no. 11, pp. 805–812, Nov. 2018, doi: 10.1007/S12275-018-8278-X.

A. Kaneko, Y. Morishita, K. Tsukada, T. Taniguchi, and T. Asai, "Post-genomic approach based discovery of alkylresorcinols from a cricket-associated fungus, *Penicillium soppi*," *Org. Biomol. Chem.*, vol. 17, no. 21, pp. 5239–5243, May 2019, doi: 10.1039/C9OB00807A.

D. Ramakrishnan *et al.*, "Molecular characterization of two alkylresorcylic acid synthases from Sordariomycetes fungi," *Enzyme Microb. Technol.*, vol. 115, pp. 16–22, Aug. 2018, doi: 10.1016/J.ENZMICTEC.2018.04.006.

G. Manoharan *et al.*, "Identification and characterization of type III polyketide synthase genes from culturable endophytes of ethnomedicinal plants," *Enzyme Microb. Technol.*, vol.

131, p. 109396, Dec. 2019, doi: 10.1016/J.ENZMICTEC.2019.109396.

L. Martinelli *et al.*, "Identification and Characterization of a New Type III Polyketide Synthase from a Marine Yeast, *Naganishia uzbekistanensis*," *Mar. Drugs*, vol. 18, no. 12, Dec. 2020, doi: 10.3390/MD18120637.

J. L. Ferrer, J. M. Jez, M. E. Bowman, R. A. Dixon, and J. P. Noel, "Structure of chalcone synthase and the molecular basis of plant polyketide biosynthesis," *Nat. Struct. Biol.*, vol. 6, no. 8, pp. 775–784, 1999, doi: 10.1038/11553.

N. B. Paniego, K. W. M. Zuurbier, S. Y. Fung, R. Van Der Heijden, J. J. C. Scheffer, and R. Verpoorte, "Phlorisovalerophenone synthase, a novel polyketide synthase from hop (*Humulus lupulus* L.) cones," *Eur. J. Biochem.*, vol. 262, no. 2, pp. 612–616, Jun. 1999, doi: 10.1046/J.1432-1327.1999.00444.X.

Y. Okada and K. Ito, "Cloning and analysis of valerophenone synthase gene expressed specifically in lupulin gland of hop (*Humulus lupulus* L.)," *Biosci. Biotechnol. Biochem.*, vol. 65, no. 1, pp. 150–155, Jan. 2001, doi: 10.1271/BBB.65.150.

K. Wanibuchi *et al.*, "An acridone-producing novel multifunctional type III polyketide synthase from *Huperzia serrata*," *FEBS J.*, vol. 274, no. 4, pp. 1073–1082, Feb. 2007, doi: 10.1111/J.1742-4658.2007.05656.X.

S. Schanz, G. Schrőder, and J. Schröder, "Stilbene synthase from Scots pine (*Pinus sylvestris*)," *FEBS Lett.*, vol. 313, no. 1, pp. 71–74, Nov. 1992, doi: 10.1016/0014-5793(92)81187-Q.

Y. Shomura *et al.*, "Crystal structure of stilbene synthase from *Arachis hypogaea*," *Proteins Struct. Funct. Bioinforma.*, vol. 60, no. 4, pp. 803–806, Sep. 2005, doi: 10.1002/PROT.20584.

R. Preisig-Müller, P. Gnau, and H. Kindl, "The inducible 9, 10-dihydrophenanthrene pathway: Characterization and expression of bibenzyl synthase and S-adenosylhomocysteine hydrolase," *Arch. Biochem. Biophys.*, vol. 317, no. 1, pp. 201–207, 1995, doi: 10.1006/abbi.1995.1154.

F. Taura *et al.*, "A novel class of plant type III polyketide synthase involved in orsellinic acid biosynthesis from *Rhododendron dauricum*," *Front. Plant Sci.*, vol. 7, no. September2016, p. 225095, Sep. 2016, doi: 10.3389/FPLS.2016.01452/BIBTEX.

F. Taura *et al.*, "Characterization of olivetol synthase, a polyketide synthase putatively involved in cannabinoid biosynthetic pathway," *FEBS Lett.*, vol. 583, no. 12, pp. 2061–2066, Jun. 2009, doi: 10.1016/J.FEBSLET.2009.05.024.

S. Eckermann *et al.*, "New pathway to polyketides in plants," *Nat. 1998 3966709*, vol. 396, no. 6709, pp. 387–390, Nov. 1998, doi: 10.1038/24652.

J. Schröder *et al.*, "Plant polyketide synthases: a chalcone synthase-type enzyme which performs a condensation reaction with methylmalonyl-CoA in the biosynthesis of C-methylated chalcones," *Biochemistry*, vol. 37, no. 23, pp. 8417–8425, Jun. 1998, doi: 10.1021/BI980204G.

Y. Mizuuchi, Y. Shimokawa, K. Wanibuchi, H. Noguchi, and I. Abe, "Structure function analysis of novel type III polyketide synthases from *Arabidopsis thaliana*," *Biol. Pharm. Bull.*, vol. 31, no. 12, pp. 2205–2210, 2008, doi: 10.1248/BPB.31.2205.

Y. Katsuyama, T. Kita, N. Funa, and S. Horinouchi, "Curcuminoid Biosynthesis by Two Type III Polyketide Synthases in the Herb *Curcuma longa*," *J. Biol. Chem.*, vol. 284, no. 17, p. 11160, Apr. 2009, doi: 10.1074/JBC.M900070200.

M. S. Resmi, P. Verma, R. S. Gokhale, and E. V. Soniya, "Identification and characterization of a type III polyketide synthase involved in quinolone alkaloid biosynthesis from *Aegle marmelos* Correa," *J. Biol. Chem.*, vol. 288, no. 10, pp. 7271–7281, Mar. 2013, doi: 10.1074/JBC.M112.429886.

I. Abe, Y. Utsumi, S. Oguro, and H. Noguchi, "The first plant type III polyketide synthase that catalyzes formation of aromatic heptaketide," *FEBS Lett.*, vol. 562, no. 1–3, pp. 171–176, Mar. 2004, doi: 10.1016/S0014-5793(04)00230-3.

I. Abe, Y. Utsumi, S. Oguro, H. Morita, Y. Sano, and H. Noguchi, "A plant type III polyketide synthase that produces pentaketide chromone," *J. Am. Chem. Soc.*, vol. 127, no. 5, pp. 1362–1363, Feb. 2005, doi: 10.1021/JA0431206.

M. Targońska-Karasek *et al.*, "Characteristic of the gene candidate SecARS encoding alkylresorcinol synthase in Secale," *Mol. Biol. Rep.*, vol. 50, no. 10, p. 8373, Oct. 2023, doi: 10.1007/S11033-023-08684-Y.

L. Meslet-Cladière *et al.*, "Structure/function analysis of a type iii polyketide synthase in the brown alga *Ectocarpus siliculosus* reveals a biochemical pathway in phlorotannin monomer biosynthesis," *Plant Cell*, vol. 25, no. 8, pp. 3089–3103, 2013, doi: 10.1105/TPC.113.111336.

H. Baharum *et al.*, "Molecular Cloning, Modeling, and Site-Directed Mutagenesis of Type III Polyketide Synthase from *Sargassum binderi* (Phaeophyta)," *Mar. Biotechnol.*, vol. 13, no. 5, pp. 845–856, Oct. 2011, doi: 10.1007/S10126-010-9344-5/FIGURES/6.

D. S. Zhao *et al.*, "A Type III Polyketide Synthase (SfuPKS1) Isolated from the Edible Seaweed *Sargassum fusiforme* Exhibits Broad Substrate and Catalysis Specificity," *J. Agric. Food Chem.*, vol. 69, no. 48, pp. 14643–14649, Dec. 2021, doi: 10.1021/ACS.JAFC.1C05868/SUPPL_FILE/JF1C05868_SI_001.PDF.

M. B. Austin *et al.*, "Biosynthesis of *Dictyostelium discoideum* differentiation-inducing factor by a hybrid type I fatty acid-type III polyketide synthase," *Nat. Chem. Biol.*, vol. 2, no. 9, pp. 494–502, 2006, doi: 10.1038/NCHEMBIO811.
N. Thenmozhi Kulasekaran, S. H. Sankara Subramanian, M. L. Thilakam, D. Gopal, J. K. Lee, and J. Marimuthu, "Functional analysis of a putative type III polyketide synthase from deep-sea sediment metagenome," *J. Biosci. Bioeng.*, Feb. 2024, doi: 10.1016/J.JBIOSC.2023.12.019.
R. J. Heath and C. O. Rock, "Inhibition of β-ketoacyl-acyl carrier protein synthase III (FabH) by acyl-acyl carrier protein in Escherichia coli," *J. Biol. Chem.*, vol. 271, no. 18, pp. 10996–11000, 1996, doi: 10.1074/JBC.271.18.10996.
K. H. Choi, L. Kremer, G. S. Besra, and C. O. Rock, "Identification and substrate specificity of β-Ketoacyl (Acyl carrier protein) synthase III (mtFabH) from *Mycobacterium tuberculosis*," *J. Biol. Chem.*, vol. 275, no. 36, pp. 28201–28207, Sep. 2000, doi: 10.1074/JBC.M003241200.
L. Han, S. Lobo, and K. A. Reynolds, "Characterization of β-ketoacyl-acyl carrier protein synthase III from *Streptomyces glaucescens* and its role in initiation of fatty acid biosynthesis," *J. Bacteriol.*, vol. 180, no. 17, pp. 4481–4486, 1998, doi: 10.1128/JB.180.17.4481-4486.1998.
K. H. Choi, R. J. Heath, and C. O. Rock, "beta-ketoacyl-acyl carrier protein synthase III (FabH) is a determining factor in branched-chain fatty acid biosynthesis," *J. Bacteriol.*, vol. 182, no. 2, pp. 365–370, Jan. 2000, doi: 10.1128/JB.182.2.365-370.2000.
M. McClelland *et al.*, "Complete genome sequence of *Salmonella enterica* serovar Typhimurium LT2," *Nat. 2001 4136858*, vol. 413, no. 6858, pp. 852–856, Oct. 2001, doi: 10.1038/35101614.
H. Takami *et al.*, "Thermoadaptation trait revealed by the genome sequence of thermophilic *Geobacillus kaustophilus*," *Nucleic Acids Res.*, vol. 32, no. 21, pp. 6292–6303, 2004, doi: 10.1093/NAR/GKH970.
W. Qian *et al.*, "Comparative and functional genomic analyses of the pathogenicity of phytopathogen *Xanthomonas campestris* pv. campestris," *Genome Res.*, vol. 15, no. 6, pp. 757–767, Jun. 2005, doi: 10.1101/GR.3378705.
T. Marti, Z. Hu, N. L. Pohl, A. N. Shah, and C. Khosla, "Cloning, nucleotide sequence, and heterologous expression of the biosynthetic gene cluster for R1128, a non-steroidal estrogen receptor antagonist. Insights into an unusual priming mechanism," *J. Biol. Chem.*, vol. 275, no. 43, pp. 33443–33448, Oct. 2000, doi: 10.1074/JBC.M006766200.
A. Grimm, K. Madduri, A. Ali, and C. R. Hutchinson, "Characterization of the *Streptomyces peucetius* ATCC 29050 genes encoding doxorubicin polyketide synthase," *Gene*, vol. 151, no. 1–2, pp. 1–10, Dec. 1994, doi: 10.1016/0378-1119(94)90625-4.
