# Supplementary material for: A Baltic Sea microbiome-derived Type III polyketide synthase produces host-protective alkylresorcinols in E. coli
Source: PLoS One. 2026 Nov 3;21(11):e0361965. doi: 10.1371/journal.pone.0361965 (PMC13730376; doi:10.1371/journal.pone.0361965)
Supplement: Supplementary file 4 [file pone.0361965.s004.pdf]

S4 Table. Complete GC-MS peak list of the Fast Blue B–positive fraction 14 from cell-associated products of *E. coli*/ArsMB.

#	t_R (min)	Identification	Match (%)	Area (%)
1	15.27	Hexadecanoic acid, methyl ester	92.9	0.256
2	15.45	Palmitoleic acid	95.0	0.494
3	15.79	*n*-Hexadecanoic acid (palmitic acid)	98.3	36.887
4	16.33	*cis*-10-Heptadecenoic acid, methyl ester	95.5	0.398
5	16.84	C17 monoenoic / cyclopropane fatty acid	97.9	33.749
6	17.31	Unidentified	—	0.192
7	17.42	13-Octadecenoic acid, methyl ester	81.1	0.061
8	17.84	*cis*-13-Octadecenoic acid	97.1	4.271
9	18.02	Octadecanoic acid	94.2	0.448
10	18.63	*cis*-10-Nonadecenoic acid, methyl ester	95.4	0.242
11	19.06	C19 monoenoic / cyclopropane fatty acid	97.9	16.527
**12**	**22.42**	**5-Tridecylbenzene-1,3-diol (ars2)**	**90.3**	**0.035**
13	24.18	Unidentified	—	0.084
**14**	**24.33**	**5-Pentadecylbenzene-1,3-diol (ars1)**	**87.1**	**1.060**
15	42.07	Unidentified	—	2.126
	—	Process-derived background (5 peaks)	—	3.170
		**Total**		**100.0**

Peak areas are percentages of the total ion chromatogram. Identifications are given as returned by the NIST 14 library. Monounsaturated and cyclopropane fatty acids of the same carbon number are isobaric ($C_nH_{2n-2}O_2$) and are not distinguished by electron-ionization mass spectrometry of the free acids. Methyl esters are attributable to the methanol used for sample dissolution and column elution. Process-derived background comprises phthalate and isophthalate esters, a fumarate diester and an antioxidant degradation product, attributable to solvents, plasticware and column bleed. The alkylresorcinols **ars1** and **ars2** are shown in bold.
