# Supplementary material for: A Baltic Sea microbiome-derived Type III polyketide synthase produces host-protective alkylresorcinols in E. coli
Source: PLoS One. 2026 Nov 3;21(11):e0361965. doi: 10.1371/journal.pone.0361965 (PMC13730376; doi:10.1371/journal.pone.0361965)
Supplement: Supplementary file 5 [file pone.0361965.s005.pdf]

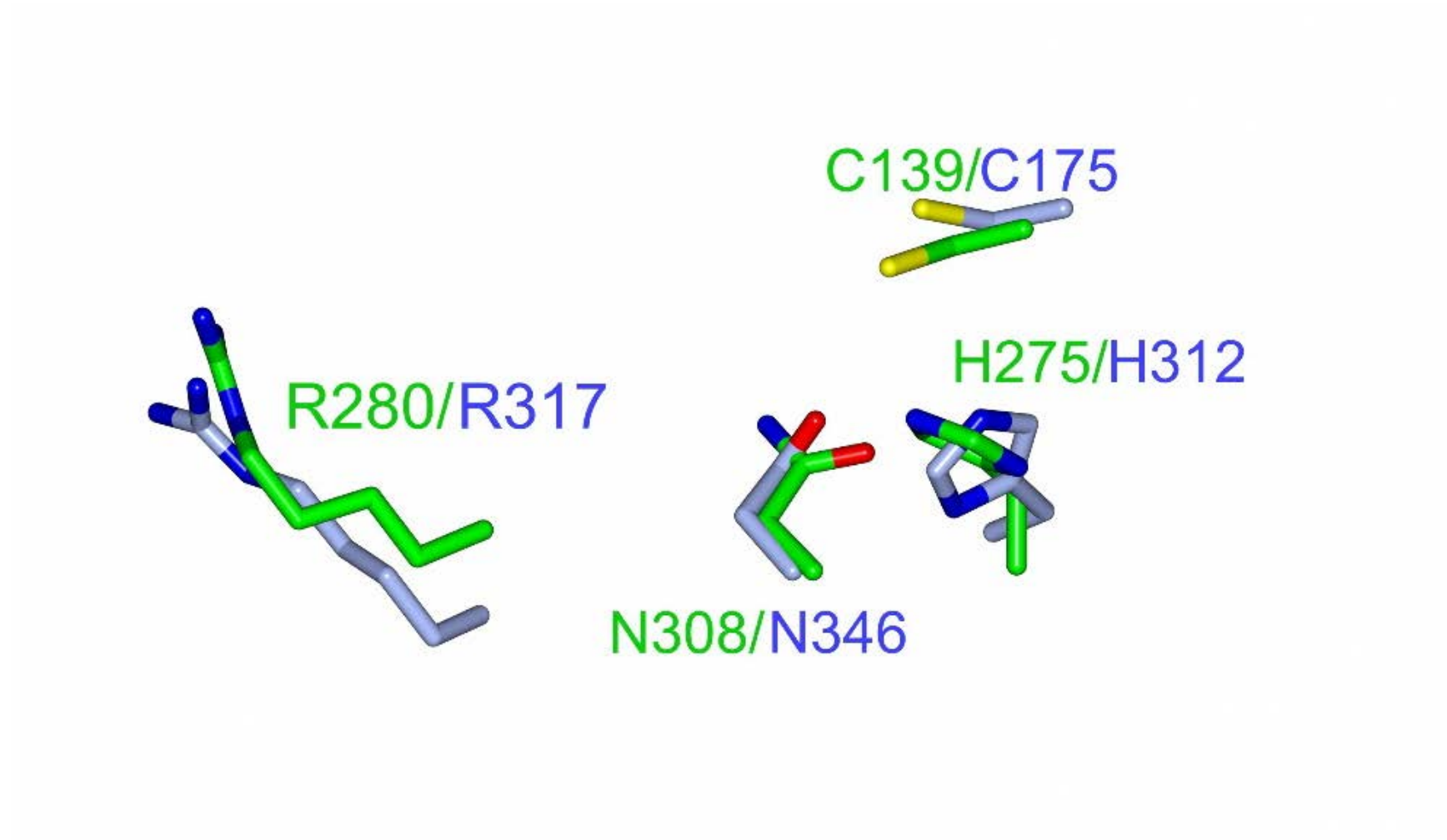

S5 Fig. Superposition of the catalytic triad and the residues R280 and R317 in ArsMB and GCS. Green legends and green carbon backbone correspond to ArsMB. Blue backbone and legends correspond to GCS (PDB: 3V7I). R317 in GCS is a key residue for Acyl-CoA/ACP binding.
