# Supplementary material for: A Baltic Sea microbiome-derived Type III polyketide synthase produces host-protective alkylresorcinols in E. coli
Source: PLoS One. 2026 Nov 3;21(11):e0361965. doi: 10.1371/journal.pone.0361965 (PMC13730376; doi:10.1371/journal.pone.0361965)
Supplement: Supplementary file 6 [file pone.0361965.s006.pdf]

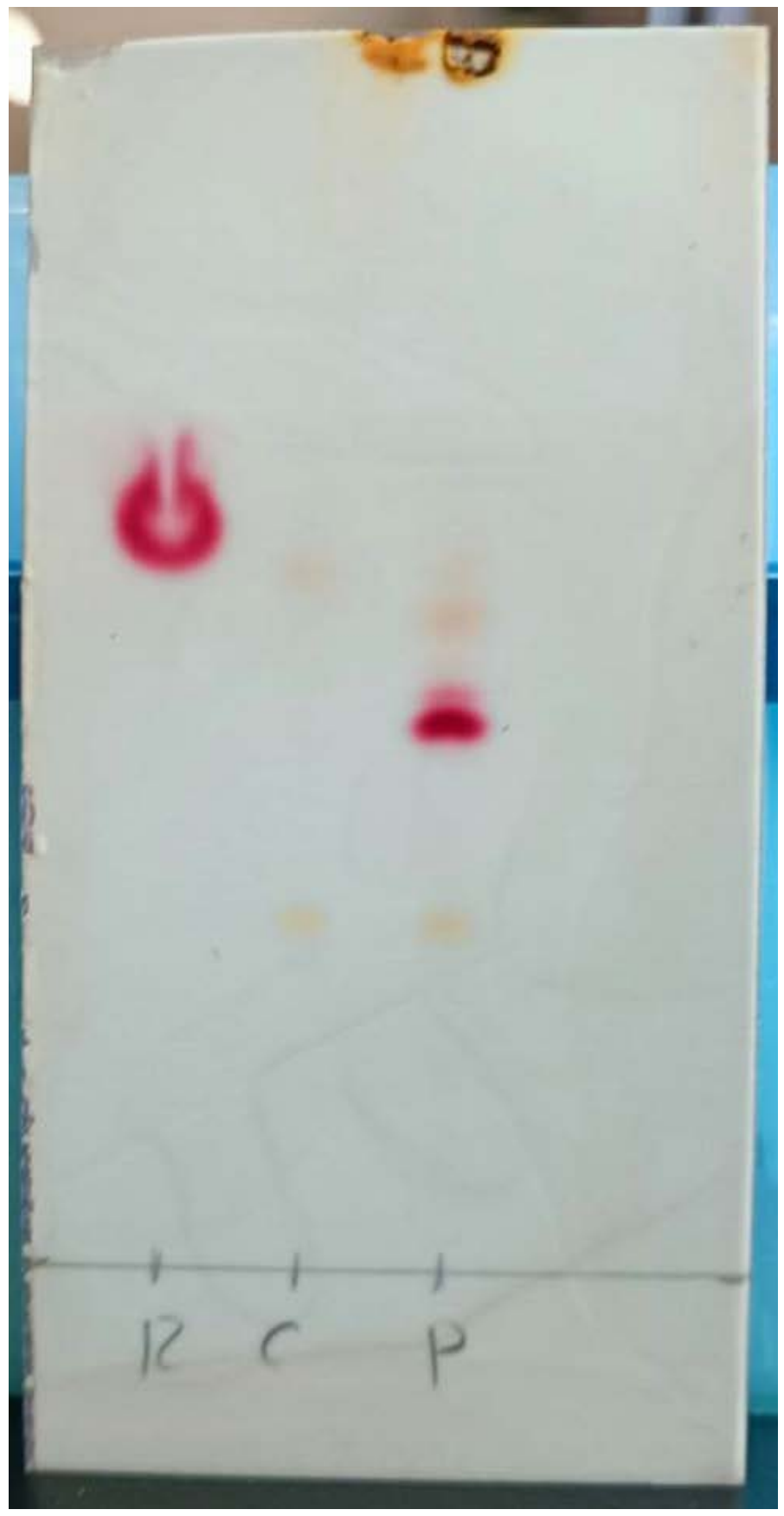

S6 Fig. TLC analysis of ethyl acetate extracts from *E. coli*/pET22b(+) and *E. coli*/ArsMB biomass. Extracts were separated on reverse-phase silica gel 60 RP-18 F_{254S}, developed with $CHCl_3$/MeOH (30:70, v/v) and stained with Fast Blue B reagent. Lane R, resorcinol standard; lane C, concentrated ethyl acetate extracts from *E. coli*/pET22b biomass; lane P, concentrated ethyl acetate extracts from *E. coli*/ArsMB biomass. The Fast Blue B-positive magenta spots appear in the resorcinol standard and the biomass extract from ArsMB-expressing strain. The image was cropped and uniformly adjusted for brightness and contrast; no other processing was applied.
