# Supplementary material for: A Baltic Sea microbiome-derived Type III polyketide synthase produces host-protective alkylresorcinols in E. coli
Source: PLoS One. 2026 Nov 3;21(11):e0361965. doi: 10.1371/journal.pone.0361965 (PMC13730376; doi:10.1371/journal.pone.0361965)
Supplement: Supplementary file 8 [file pone.0361965.s008.pdf]

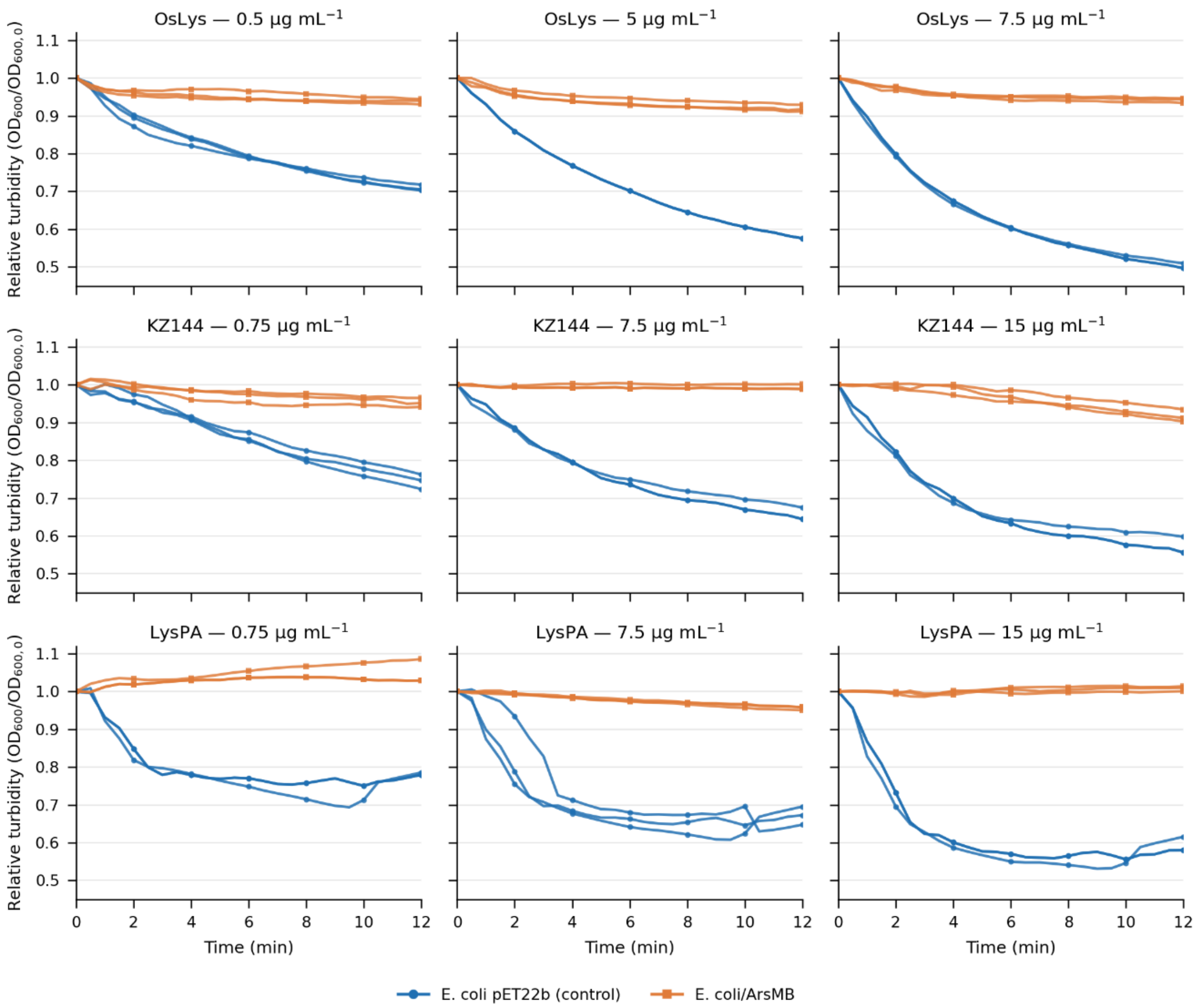

S8 Fig. Individual replicate traces of the muralytic assays. Turbidity of chloroform-sensitized cells of *E. coli*/pET-22b(+) (control, blue circles) and *E. coli*/ArsMB (orange squares), normalised to the initial OD_{600nm} of each well, after addition of OsLys, LysKZ144 or LysPA26 at the three concentrations indicated. All three independent biological replicates are plotted individually; traces of the same colour that appear superimposed differ by less than the line width.
