# Supplementary material for: A Baltic Sea microbiome-derived Type III polyketide synthase produces host-protective alkylresorcinols in E. coli
Source: PLoS One. 2026 Nov 3;21(11):e0361965. doi: 10.1371/journal.pone.0361965 (PMC13730376; doi:10.1371/journal.pone.0361965)
Supplement: Supplementary file 9 [file pone.0361965.s009.pdf]

S9 Table. Strain contrasts on the derived parameters of the H_2O_2 and muralytic assays shown in Fig 6. Parameters obtained for every individual well (AUC, $\Delta OD_{600,16h}$, recovery lag λ, lysis constant k, residual plateau P, initial rate and lysis AUC), together with the two-way ANOVA tables, partial η^2 values, and the strain contrasts at each concentration with their differences of means, 95 % confidence intervals, Hedges' g and Holm-adjusted p values.

S9a Table

[H_2O_2] mM	AUC ars(−)	AUC ars(+)	Δ AUC [IC 95 %]	p (Holm)	g
0	5.52 ± 0.06	5.38 ± 0.04	−0.14 [−0.43, 0.15]	0.42	−2.1
0.2	0.11 ± 0.11	1.75 ± 0.29	+1.64 [1.01, 2.26]	0.020	6.0
0.4	−0.05 ± 0.01	0.24 ± 0.02	+0.30 [0.26, 0.34]	0.00096	16.5
0.7	−0.08 ± 0.02	−0.07 ± 0.03	+0.01 [−0.06, 0.08]	1.00	0.3
1.0	−0.35 ± 0.02	−0.33 ± 0.28	+0.03 [−0.66, 0.71]	1.00	0.1

AUC in $\Delta OD_{600}\cdot h$, mean ± SD. by two-way ANOVA: strain $F_{1,18} = 56.4$, $\eta^2p = 0.76$; [H_2O_2] $F_{4,18} = 1474$, $\eta^2p = 0.997$; interaction $F_{4,18} = 38.4$, $\eta^2p = 0.89$ (all $p < 0.0001$).

S9b Table

Enzyme	µg	AUC ars(−)	AUC ars(+)	Δ [IC 95 %]	p (Holm)	g
LysKZ144	0.5	2.30 ± 0.05	0.57 ± 0.10	1.73 [1.51, 1.95]	0.00081	17.3
LysKZ144	5.0	3.28 ± 0.00	0.71 ± 0.10	2.57 [2.32, 2.83]	0.0016	28.0
LysKZ144	7.5	4.22 ± 0.00	0.54 ± 0.05	3.69 [3.57, 3.80]	0.00027	88.8
LysPA26	0.75	2.48 ± 0.12	−0.44 ± 0.17	2.92 [2.57, 3.27]	0.00027	16.0
LysPA26	7.5	3.38 ± 0.32	0.27 ± 0.03	3.11 [2.32, 3.90]	0.0033	10.8
LysPA26	15.0	4.45 ± 0.09	−0.02 ± 0.04	4.47 [4.29, 4.65]	0.000046	52.9
OsLys	0.75	1.63 ± 0.09	0.31 ± 0.14	1.31 [1.02, 1.60]	0.0016	9.0
OsLys	7.5	2.77 ± 0.08	0.06 ± 0.06	2.71 [2.56, 2.87]	0.000018	32.2
OsLys	12.5	3.78 ± 0.07	0.41 ± 0.13	3.37 [3.10, 3.64]	0.00027	26.2
