# Supplementary material for: A Baltic Sea microbiome-derived Type III polyketide synthase produces host-protective alkylresorcinols in E. coli
Source: PLoS One. 2026 Nov 3;21(11):e0361965. doi: 10.1371/journal.pone.0361965 (PMC13730376; doi:10.1371/journal.pone.0361965)
Supplement: Supplementary file 10 [file pone.0361965.s010.pdf]

S10 Table. Per-well derived parameters and statistical analysis of the assays shown in Fig 6.

Parameters obtained for every individual well of the H_2O_2 exposure assay (Fig 6A) and of the turbidity-reduction assay with endolysins (Fig 6B), together with the corresponding two-way ANOVA tables, partial η^2 values and strain contrasts. *E. coli* arsMB (−) denotes the non-producing control strain carrying the empty pET-22b(+) vector and *E. coli* arsMB (+) the ArsMB-producing strain. Statistical inference was performed on these per-well summary parameters rather than on individual time points, so that each well contributes a single independent observation; strain contrasts were evaluated by Welch's *t*-test with Holm–Bonferroni correction across the whole family of concentrations of each assay, and effect sizes are given as the difference between means with its 95 % confidence interval and as Hedges' *g*.

Table A. Per-well parameters of the H_2O_2 exposure assay (Fig 6A).

Strain	$[H_2O_2]$ (mM)	Well	AUC 0–16 h (ΔOD600·h)	ΔOD600 at 16 h	λ (h)	Independent trace
arsMB (+)	0	1	5.342	0.578	2.45	Yes
arsMB (+)	0	2	5.380	0.585	2.57	Yes
arsMB (+)	0	3	5.418	0.596	2.57	Yes
arsMB (+)	0.2	1	2.061	0.426	7.82	Yes
arsMB (+)	0.2	2	1.489	0.370	9.22	Yes
arsMB (+)	0.2	3	1.692	0.401	8.75	Yes
arsMB (+)	0.25	1	1.317	0.331	9.80	Yes
arsMB (+)	0.25	2	1.314	0.330	9.80	Yes
arsMB (+)	0.25	3	1.324	0.333	9.68	Yes
arsMB (+)	0.3	1	0.868	0.253	11.08	Yes
arsMB (+)	0.3	2	0.866	0.252	11.08	Yes
arsMB (+)	0.3	3	0.871	0.254	10.97	Yes
arsMB (+)	0.35	1	0.486	0.179	12.48	Yes
arsMB (+)	0.35	2	0.481	0.177	12.60	Yes
arsMB (+)	0.35	3	0.487	0.180	12.48	Yes
arsMB (+)	0.4	1	0.266	0.142	13.77	Yes

Strain	$[H_2O_2]$ (mM)	Well	AUC 0–16 h (ΔOD600·h)	ΔOD600 at 16 h	λ (h)	Independent trace
arsMB (+)	0.4	2	0.232	0.127	14.00	Yes
arsMB (+)	0.4	3	0.236	0.128	14.00	Yes
arsMB (+)	0.7	1	-0.112	-0.005	n.r.	Yes
arsMB (+)	0.7	2	-0.054	0.003	n.r.	Yes
arsMB (+)	0.7	3	-0.049	0.003	n.r.	Yes
arsMB (+)	1	1	-0.015	-0.020	n.r.	Yes
arsMB (+)	1	2	-0.412	-0.060	n.r.	Yes
arsMB (+)	1	3	-0.550	-0.050	n.r.	Yes
arsMB (-)	0	2	5.562	0.602	2.33	Yes
arsMB (-)	0	3	5.475	0.599	2.45	Yes
arsMB (-)	0.2	1	0.059	0.054	15.87	Yes
arsMB (-)	0.2	2	0.033	0.056	15.87	Yes
arsMB (-)	0.2	3	0.239	0.135	13.88	Yes
arsMB (-)	0.25	1	-0.049	0.001	n.r.	Yes
arsMB (-)	0.25	2	-0.028	0.003	n.r.	Yes
arsMB (-)	0.25	3	-0.050	0.001	n.r.	No
arsMB (-)	0.3	1	-0.069	-0.002	n.r.	Yes
arsMB (-)	0.3	2	-0.073	-0.002	n.r.	Yes
arsMB (-)	0.3	3	-0.067	-0.002	n.r.	Yes
arsMB (-)	0.35	1	-0.062	-0.003	n.r.	Yes
arsMB (-)	0.35	2	-0.062	-0.003	n.r.	Yes
arsMB (-)	0.35	3	-0.065	-0.004	n.r.	Yes
arsMB (-)	0.4	1	-0.051	-0.002	n.r.	Yes
arsMB (-)	0.4	2	-0.065	-0.002	n.r.	Yes
arsMB (-)	0.4	3	-0.049	0.002	n.r.	Yes
arsMB (-)	0.7	1	-0.091	-0.008	n.r.	Yes

Strain	$[H_2O_2]$ (mM)	Well	AUC 0–16 h (ΔOD600·h)	ΔOD600 at 16 h	λ (h)	Independent trace
arsMB (-)	0.7	2	-0.089	-0.007	n.r.	Yes
arsMB (-)	0.7	3	-0.062	-0.005	n.r.	Yes
arsMB (-)	1	1	-0.364	-0.040	n.r.	Yes
arsMB (-)	1	3	-0.338	-0.040	n.r.	Yes

λ, recovery lag, defined as the time required to reach $\Delta OD_{600} = 0.05$; n.r., the threshold was not reached within the 16 h of the assay. The 0 mM and 1.0 mM conditions of the ars(−) strain contain two wells only.

Table B. H_2O_2 assay: descriptive statistics and strain contrasts.

$[H_2O_2]$ (mM)	Parameter	arsMB (−)	arsMB (+)	Difference [95 % CI]	t	df	p (Holm)	Hedges' g
0	AUC 0–16 h	5.52 ± 0.06	5.38 ± 0.04	−0.14 [−0.43, 0.15]	−2.85	1.52	0.42	−2.1
0.2	AUC 0–16 h	0.11 ± 0.11	1.75 ± 0.29	+1.64 [1.01, 2.26]	9.12	2.58	0.020	6.0
0.4	AUC 0–16 h	−0.05 ± 0.01	0.24 ± 0.02	+0.30 [0.26, 0.34]	25.26	2.85	9.6×10^{-4}	16.5
0.7	AUC 0–16 h	−0.08 ± 0.02	−0.07 ± 0.03	+0.01 [−0.06, 0.08]	0.42	2.82	1.00	0.3
1.0	AUC 0–16 h	−0.35 ± 0.02	−0.33 ± 0.28	+0.03 [−0.66, 0.71]	0.16	2.03	1.00	0.1
0	ΔOD600 at 16 h	0.601 ± 0.002	0.586 ± 0.009	−0.014 [−0.035, 0.007]	−2.60	2.31	0.32	−1.4
0.2	ΔOD600 at 16 h	0.082 ± 0.046	0.399 ± 0.028	+0.317 [0.223, 0.412]	10.17	3.30	5.3×10^{-3}	6.6
0.4	ΔOD600 at 16 h	−0.001 ± 0.002	0.132 ± 0.008	+0.133 [0.114, 0.152]	26.48	2.30	3.3×10^{-3}	17.3
0.7	ΔOD600 at 16 h	−0.007 ± 0.002	0.000 ± 0.005	+0.007 [−0.003, 0.017]	2.49	2.43	0.32	1.6
1.0	ΔOD600 at 16 h	−0.040 ± 0.000	−0.043 ± 0.021	−0.003 [−0.055, 0.048]	−0.28	2.00	0.81	−0.1
0	λ (h)	2.39 ± 0.08	2.53 ± 0.07	+0.14 [−0.18, 0.45]	1.64	2.83	0.20	1.4
0.2	λ (h)	15.21 ± 1.15	8.59 ± 0.71	−6.61 [−8.95, −4.27]	−8.48	3.33	2.2×10^{-3}	−5.6

Values are mean ± SD of three independent wells, except for the ars(−) strain at 0 and 1.0 mM (two wells). At $H_2O_2 \geq 0.25$ mM the ars(−) strain never reached the λ threshold, so no contrast could be computed for that parameter.

Table C. H_2O_2 assay: two-way ANOVA (strain × $[H_2O_2]$).

Response	Source	SS	df	F	p	Partial η^2
AUC 0–16 h	$[H_2O_2]$	117.377	4	1474.05	< 0.0001	0.997
	Strain	1.122	1	56.37	< 0.0001	0.758
	Strain × $[H_2O_2]$	3.054	4	38.35	< 0.0001	0.895
	Residual	0.358	18	—	—	—
ΔOD600 at 16 h	$[H_2O_2]$	1.366	4	868.54	< 0.0001	0.995
	Strain	0.064	1	163.01	< 0.0001	0.901
	Strain × $[H_2O_2]$	0.114	4	72.37	< 0.0001	0.941
	Residual	0.007	18	—	—	—

Table D. H_2O_2 assay: linear mixed-effects model of the complete time courses.

Fixed effect	β	95 % CI	z	p
Intercept	−0.031	−0.049 to −0.014	−3.52	< 0.001
Strain [arsMB (+)]	−0.138	−0.162 to −0.113	−10.98	< 0.0001
Time	0.006	0.001 to 0.011	2.54	0.011
Strain × time	0.041	0.034 to 0.047	11.90	< 0.0001
$[H_2O_2]$	0.071	0.016 to 0.126	2.55	0.011
Strain × $[H_2O_2]$	0.288	0.210 to 0.365	7.25	< 0.0001
Time × $[H_2O_2]$	−0.016	−0.030 to −0.001	−2.04	0.041
Strain × time × $[H_2O_2]$	−0.088	−0.109 to −0.067	−8.19	< 0.0001

Model: ΔOD_{600} ~ strain × time × $[H_2O_2]$, with a random intercept and a random slope per well; 1,653 readings from 12 wells (0.2 and 0.4 mM). β for the interaction terms is expressed in $\Delta OD_{600}\ h^{-1}$.

Table E. Per-well kinetic parameters of the turbidity-reduction assay with endolysins (Fig 6B).

Endolysin	Enzyme (µg)	Strain	Well	k (min−1)	P	v0 (min−1)	Initial rate (min−1)	Lysis AUC 0–12 min	Rel. turbidity 12 min	R^2	Indep.
LysKZ144	0.5	arsMB (+)	1	0.412	0.954	0.0189	0.0157	0.448	0.944	0.595	Yes
LysKZ144	0.5	arsMB (+)	2	0.367	0.934	0.0242	0.0181	0.618	0.930	0.944	Yes
LysKZ144	0.5	arsMB (+)	3	0.864	0.942	0.0505	0.0217	0.635	0.940	0.962	Yes
LysKZ144	0.5	arsMB (-)	1	0.260	0.718	0.0734	0.0674	2.351	0.718	0.986	Yes
LysKZ144	0.5	arsMB (-)	2	0.141	0.636	0.0512	0.0476	2.262	0.703	0.999	Yes
LysKZ144	0.5	arsMB (-)	3	0.159	0.656	0.0546	0.0555	2.284	0.705	0.997	Yes
LysKZ144	5	arsMB (+)	1	0.344	0.916	0.0290	0.0224	0.775	0.912	0.972	Yes
LysKZ144	5	arsMB (+)	2	0.273	0.930	0.0190	0.0187	0.588	0.929	0.982	Yes
LysKZ144	5	arsMB (+)	3	0.359	0.918	0.0295	0.0225	0.759	0.918	0.996	Yes
LysKZ144	5	arsMB (-)	1	0.157	0.503	0.0780	0.0703	3.282	0.576	0.999	Yes
LysKZ144	5	arsMB (-)	2	0.157	0.503	0.0780	0.0703	3.282	0.576	0.999	Yes
LysKZ144	5	arsMB (-)	3	0.157	0.503	0.0780	0.0703	3.282	0.576	0.999	Yes
LysKZ144	7.5	arsMB (+)	1	0.364	0.947	0.0192	0.0127	0.488	0.946	0.983	Yes
LysKZ144	7.5	arsMB (+)	2	0.282	0.932	0.0192	0.0120	0.582	0.933	0.990	Yes
LysKZ144	7.5	arsMB (+)	3	0.480	0.945	0.0263	0.0183	0.542	0.943	0.982	Yes
LysKZ144	7.5	arsMB (-)	1	0.239	0.475	0.1254	0.1004	4.224	0.497	0.999	Yes
LysKZ144	7.5	arsMB (-)	2	0.239	0.475	0.1254	0.1004	4.224	0.497	0.999	No
LysKZ144	7.5	arsMB (-)	3	0.267	0.497	0.1343	0.1034	4.226	0.510	0.999	Yes
LysPA26	0.75	arsMB (+)	1	0.139	1.100	-0.0139	-0.0163	-0.631	1.086	0.915	Yes
LysPA26	0.75	arsMB (+)	2	0.458	1.035	-0.0159	-0.0118	-0.339	1.029	0.886	Yes
LysPA26	0.75	arsMB (+)	3	0.458	1.035	-0.0159	-0.0118	-0.339	1.029	0.886	No
LysPA26	0.75	arsMB (-)	1	0.501	0.756	0.1221	0.0792	2.416	0.780	0.943	Yes
LysPA26	0.75	arsMB (-)	2	0.501	0.756	0.1221	0.0792	2.416	0.780	0.943	No

Endolysin	Enzyme (µg)	Strain	Well	k (min−1)	P	v0 (min−1)	Initial rate (min−1)	Lysis AUC 0–12 min	Rel. turbidity 12 min	R^2	Indep.
LysPA26	0.75	arsMB (-)	3	0.446	0.730	0.1203	0.0990	2.623	0.786	0.896	Yes
LysPA26	7.5	arsMB (+)	1	0.007	0.376	0.0044	0.0040	0.311	0.951	0.996	Yes
LysPA26	7.5	arsMB (+)	2	0.052	0.911	0.0046	0.0026	0.270	0.959	0.985	Yes
LysPA26	7.5	arsMB (+)	3	0.004	0.002	0.0035	0.0012	0.241	0.958	0.977	Yes
LysPA26	7.5	arsMB (-)	1	0.226	0.606	0.0889	0.0325	3.023	0.648	0.898	Yes
LysPA26	7.5	arsMB (-)	2	0.492	0.649	0.1728	0.1090	3.475	0.672	0.963	Yes
LysPA26	7.5	arsMB (-)	3	0.514	0.636	0.1873	0.1304	3.649	0.696	0.936	Yes
LysPA26	15	arsMB (+)	1	0.011	1.100	-0.0011	0.0034	-0.054	1.014	0.441	Yes
LysPA26	15	arsMB (+)	2	1.072	0.998	0.0025	0.0007	0.026	1.001	0.054	Yes
LysPA26	15	arsMB (+)	3	0.007	1.100	-0.0007	0.0021	-0.039	1.010	0.541	Yes
LysPA26	15	arsMB (-)	1	0.496	0.556	0.2202	0.1366	4.401	0.580	0.971	Yes
LysPA26	15	arsMB (-)	2	0.496	0.556	0.2202	0.1366	4.401	0.580	0.971	No
LysPA26	15	arsMB (-)	3	0.559	0.553	0.2500	0.1592	4.550	0.616	0.951	Yes
OsLys	0.75	arsMB (+)	1	0.173	0.931	0.0120	0.0037	0.476	0.942	0.929	Yes
OsLys	0.75	arsMB (+)	2	0.018	0.815	0.0033	0.0064	0.213	0.965	0.911	Yes
OsLys	0.75	arsMB (+)	3	0.004	0.000	0.0040	0.0003	0.251	0.952	0.874	Yes
OsLys	0.75	arsMB (-)	1	0.027	0.000	0.0269	0.0090	1.679	0.724	0.975	Yes
OsLys	0.75	arsMB (-)	2	0.046	0.400	0.0279	0.0231	1.673	0.747	0.995	Yes
OsLys	0.75	arsMB (-)	3	0.035	0.304	0.0242	0.0198	1.528	0.763	0.996	Yes
OsLys	7.5	arsMB (+)	1	0.531	0.991	0.0050	0.0042	0.095	0.988	0.794	Yes
OsLys	7.5	arsMB (+)	2	0.533	0.990	0.0051	0.0042	0.095	0.989	0.849	Yes
OsLys	7.5	arsMB (+)	3	0.162	1.002	-0.0003	0.0018	-0.006	1.001	0.122	Yes
OsLys	7.5	arsMB (-)	1	0.185	0.607	0.0729	0.0565	2.818	0.644	0.998	Yes
OsLys	7.5	arsMB (-)	2	0.185	0.607	0.0729	0.0565	2.818	0.644	0.998	No
OsLys	7.5	arsMB (-)	3	0.237	0.666	0.0794	0.0567	2.688	0.675	0.997	Yes
OsLys	12.5	arsMB (+)	1	0.008	0.000	0.0080	0.0055	0.533	0.903	0.981	Yes

Endolysin	Enzyme (µg)	Strain	Well	k (min−1)	P	v0 (min−1)	Initial rate (min−1)	Lysis AUC 0–12 min	Rel. turbidity 12 min	R^2	Indep.
OsLys	12.5	arsMB (+)	2	0.007	0.000	0.0067	-0.0020	0.418	0.912	0.880	Yes
OsLys	12.5	arsMB (+)	3	0.004	0.000	0.0044	0.0029	0.274	0.934	0.834	Yes
OsLys	12.5	arsMB (-)	1	0.268	0.544	0.1220	0.0876	3.820	0.556	0.997	Yes
OsLys	12.5	arsMB (-)	2	0.268	0.544	0.1220	0.0876	3.820	0.556	0.997	No
OsLys	12.5	arsMB (-)	3	0.359	0.599	0.1439	0.0906	3.707	0.598	0.997	Yes

k, first-order lysis constant and P, residual turbidity plateau, obtained by fitting each well to $\Delta OD_{600}(t)/\Delta OD_{600}(0) = P + (1 - P)e^{-kt}$; $v_0 = k(1 - P)$; initial rate, slope over the first 2 min; lysis AUC, area above the normalised turbidity curve between 0 and 12 min. In the ArsMB-producing strain the traces are almost flat, so k and P are poorly constrained (see R^2) and only the initial rate, the lysis AUC and the relative turbidity at 12 min should be used for comparison. Wells marked "No" are not independent replicates (see Table A footnote). The assignment of each dataset to its endolysin follows the enzyme concentrations given in Materials and methods and the panels of Fig 6B.

Table F. Endolysin assay: strain contrasts on the lysis $AUC_{0–12min}$.

Endolysin	Enzyme (µg)	arsMB (−)	arsMB (+)	Residual lysis (%)	Difference [95 % CI]	t	p (Holm)	Hedges' g
LysKZ144	0.5	2.30 ± 0.05	0.57 ± 0.10	24.7	1.73 [1.51, 1.95]	26.56	8.1×10^{-4}	17.3
LysKZ144	5.0	3.28 ± 0.00	0.71 ± 0.10	21.6	2.57 [2.32, 2.83]	42.88	1.6×10^{-3}	28.0
LysKZ144	7.5	4.22 ± 0.00	0.54 ± 0.05	12.7	3.69 [3.57, 3.80]	135.95	2.7×10^{-4}	88.8
LysPA26	0.75	2.48 ± 0.12	−0.44 ± 0.17	< 0	2.92 [2.57, 3.27]	24.44	2.7×10^{-4}	16.0
LysPA26	7.5	3.38 ± 0.32	0.27 ± 0.03	8.1	3.11 [2.32, 3.90]	16.56	3.3×10^{-3}	10.8
LysPA26	15.0	4.45 ± 0.09	−0.02 ± 0.04	< 0	4.47 [4.29, 4.65]	80.91	4.6×10^{-5}	52.9
OsLys	0.75	1.63 ± 0.09	0.31 ± 0.14	19.3	1.31 [1.02, 1.60]	13.71	1.6×10^{-3}	9.0
OsLys	7.5	2.77 ± 0.08	0.06 ± 0.06	2.2	2.71 [2.56, 2.87]	49.26	1.8×10^{-5}	32.2
OsLys	12.5	3.78 ± 0.07	0.41 ± 0.13	10.8	3.37 [3.10, 3.64]	40.16	2.7×10^{-4}	26.2

Residual lysis, lysis AUC of the producing strain expressed as a percentage of that of the control strain; "< 0" indicates that the turbidity of the producing strain did not decrease at all over the 12 min of the assay. Contrasts on the relative turbidity at 12 min gave the same outcome (all Holm-adjusted $p \leq 4.6 \times 10^{-3}$).

Table G. Endolysin assay: two-way ANOVA (strain × enzyme concentration) on the lysis $AUC_{0–12min}$.

Endolysin	Source	SS	df	F	p	Partial η^2
LysKZ144	Concentration	2.726	2	317.17	< 0.0001	0.981

Endolysin	Source	SS	df	F	p	Partial η^2
	Strain	31.948	1	7434.84	< 0.0001	0.998
	Strain × concentration	2.884	2	335.53	< 0.0001	0.982
	Residual	0.052	12	—	—	—
LysPA26	Concentration	4.421	2	84.12	< 0.0001	0.933
	Strain	55.146	1	2098.38	< 0.0001	0.994
	Strain × concentration	2.154	2	40.98	< 0.0001	0.872
	Residual	0.315	12	—	—	—
OsLys	Concentration	3.851	2	199.85	< 0.0001	0.971
	Strain	27.386	1	2842.71	< 0.0001	0.996
	Strain × concentration	3.321	2	172.35	< 0.0001	0.966
	Residual	0.116	12	—	—	—

All analyses were performed in Python 3.11 (SciPy; statsmodels 0.15) and cross-checked in GraphPad Prism v8.0.1, with α = 0.05.
